# Diverse in- and output polarities and high complexity of local synaptic and non-synaptic signaling within a chemically defined class of peptidergic *Drosophila* neurons

**DOI:** 10.3389/fncir.2013.00127

**Published:** 2013-08-01

**Authors:** Gergely Karsai, Edit Pollák, Matthias Wacker, Matthias Vömel, Mareike Selcho, Gergely Berta, Ronald J. Nachman, R. Elwyn Isaac, László Molnár, Christian Wegener

**Affiliations:** ^1^Department of Comparative Anatomy and Developmental Biology, Institute of Biology, Faculty of Science, University of PécsPécs, Hungary; ^2^Neurobiology and Genetics, Biocenter, Theodor-Boveri-Institute, University of WürzburgWürzburg, Germany; ^3^Animal Physiology, Department of Biology, Philipps-UniversityMarburg, Germany; ^4^Faculty of Medicine, Department of Medical Biology, University of PécsPécs, Hungary; ^5^Areawide Pest Management Research, Southern Plains Agricultural Research Center, United States Department of Agriculture, College StationTX, USA; ^6^Faculty of Biological Sciences, School of Biology, University of LeedsLeeds, UK

**Keywords:** synaptic signaling, volume transmission, paracrine release, neuromodulation, ecdysis, bursicon, CCAP, myoinhibitory peptide

## Abstract

Peptidergic neurons are not easily integrated into current connectomics concepts, since their peptide messages can be distributed via non-synaptic paracrine signaling or volume transmission. Moreover, the polarity of peptidergic interneurons in terms of in- and out-put sites can be hard to predict and is very little explored. We describe in detail the morphology and the subcellular distribution of fluorescent vesicle/dendrite markers in CCAP neurons (N_CCAP_), a well defined set of peptidergic neurons in the *Drosophila* larva. N_CCAP_ can be divided into five morphologically distinct subsets. In contrast to other subsets, serial homologous interneurons in the ventral ganglion show a mixed localization of in- and output markers along ventral neurites that defy a classification as dendritic or axonal compartments. Ultrastructurally, these neurites contain both pre- and postsynaptic sites preferably at varicosities. A significant portion of the synaptic events are due to reciprocal synapses. Peptides are mostly non-synaptically or parasynaptically released, and dense-core vesicles and synaptic vesicle pools are typically well separated. The responsiveness of the N_CCAP_ to ecdysis-triggering hormone may be at least partly dependent on a tonic synaptic inhibition, and is independent of ecdysteroids. Our results reveal a remarkable variety and complexity of local synaptic circuitry within a chemically defined set of peptidergic neurons. Synaptic transmitter signaling as well as peptidergic paracrine signaling and volume transmission from varicosities can be main signaling modes of peptidergic interneurons depending on the subcellular region. The possibility of region-specific variable signaling modes should be taken into account in connectomic studies that aim to dissect the circuitry underlying insect behavior and physiology, in which peptidergic neurons act as important regulators.

## Introduction

Understanding neuronal connectivity in brains is a major goal in neuroscience. With recent advances in microscopy, genetically expressed marker proteins and the development of suitable software and computational power to reconstruct large high-resolution anatomical datasets in 3D [see e.g., (Briggman and Denk, 2006; Smith, 2007; Cardona et al., 2010; Mishchenko, 2011)], large scale anatomical “connectomics” studies have now become possible.

Due to their relative small number of neurons, invertebrates have since long provided valuable model systems to study the wiring of neuronal circuits [see (White et al., 1986; Clarac and Pearlstein, 2007)]. Recently, anatomical circuit reconstruction has started in the genetically amenable fruitfly *Drosophila* (e.g., Yu et al., 2010; Lai et al., 2012): neurons are computed into a standard brain, and sites of close apposition or overlap of projections in combination with the expression of tagged pre- and postsynaptic markers such as syb::GFP (Estes et al., 2000) or Dscam::GFP (Wang et al., 2004) are then interpreted to indicate synaptic contacts. Once pre- and postsynaptic compartments are identified, it is straightforward to predict the direction of information flow. Implicit in this approach is Cajal's “law of dynamic polarization” derived from the morphology of vertebrate neurons: neurons receive input onto postsynaptic dendrites and provide output via presynaptic axon terminals (Shepherd, 1987) with the in- and output compartments spatially well separated. Dendrites and axons of the usually unipolar invertebrate neurons are not clearly separated by the soma, but the common view is that the primary neurite of a typical insect neuron forms different and separated branches which act (predominantly) as dendritic input or axonal output compartments (Cardona et al., 2010). In that sense, a clear polarity and intracellular compartmentalization into dendrites, axons, and presynaptic axon terminals has indeed been demonstrated for several neuron types of the fruitfly, such as motorneurons (Sánchez-Soriano et al., 2005) and sensory neurons [see (Rolls, 2011)].

Nevertheless, it is widely acknowledged that a purely anatomical “connectomics” approach is not only essential but also over-simplistic and insufficient. It largely ignores not only variabilities in synaptic strength, but also neuromodulatory signaling (Marder, 2012), a mechanism which aptly has been named “signaling beyond the wiring diagram” (Brezina, 2010). Peptidergic interneurons are central components of neuromodulatory signaling networks, but it can be very difficult to anatomically determine their target cells which—in case of volume transmission - can be located a considerable distance away from the peptide release sites [see (Agnati et al., 1995; Fuxe et al., 2007; Van den Pol, 2012)].

A long-known fact further complicates anatomical circuit analysis: not all neurons follow Cajal's “law of dynamic polarization,” but show dendro-dendritic or axo-axonic interactions (Shepherd, 1987). Though highly variable in extent, presynaptic elements on dendritic structures or postsynaptic elements on axons are not uncommon in vertebrates [see (Shepherd, 1987)] and appear to be the rule rather than the exception in insect neurons [e.g., (Strausfeld, 1976; Watson and Burrows, 1983, 1985, 1988; Peters et al., 1986; Cardona et al., 2010; Christiansen et al., 2011)]. Especially for peptidergic insect neurons, polarity can be very difficult to predict (Nässel, 2009). Neuropeptides stored in dense-core vesicles (DCV) can be released along axons and dendrites in a parasynaptical (close but not at the active zone of a synapse) or non-synaptical fashion [see (Golding, 1994; Agnati et al., 1995; Ludwig and Leng, 2006). Both axo-axonic (e.g., Silverman et al., 1983; Guan et al., 2003) and dendro-dendritic synapses (e.g., Silverman and Witkin, 1985; Campbell et al., 2009) were found for vertebrate peptidergic neurons though they clearly do not represent the main type of synaptic connection for peptidergic neurons.

In the fruitfly, a clear spatial separation of pre- and postsynaptic compartments based on the distribution of pre- and postsynaptic markers has been implicated for both peptidergic interneurons (Hamasaka et al., 2005; Nicolaï et al., 2010) and neurosecretory cells (e.g., Santos et al., 2007). On the other hand, a co-occurrence or close apposition of pre- and postsynaptic markers on the light-microscopic level has been found for both neurohaemal release sites (Nicolaï et al., 2010) as well as peptidergic projections within the CNS (e.g., Santos et al., 2007).

Detailed high-resolution studies of the synaptology of “mixed” neurites in peptidergic interneurons are generally rare and essentially lacking for invertebrates such as insects. With respect to the current connectome mapping and circuit reconstruction efforts and the frequent use of genetic markers in *Drosophila*, there are several open questions regarding peptidergic interneurons in the fruitfly: (i) how well are in- and output sites separated or overlapping in “mixed” peptidergic neurites? (ii) where along a synaptic/volume transmission gradient are peptidergic interneurons ranging? and (iii) do ectopically expressed presynaptic and dendritic markers faithfully report in- and output compartments in peptidergic neurons? Answering these questions will be important to assess whether and how peptidergic interneurons can be fitted into current connectomic concepts in the fruitfly and other species.

We here report on a detailed anatomical study on the CNS projections of larval CCAP neurons (N_CCAP_) in *Drosophila melanogaster.* The study is designed to address the questions outlined above for a small but well defined set of peptidergic neurons that are involved in the timing and organization of ecdysis, a crucial motor behavior of arthropods (Park et al., 2003; Clark et al., 2004). N_CCAP_ form a highly conserved set of neurons throughout the insects and comprize efferent neurons as well as local and projection interneurons (Dircksen, 1994; Ewer and Truman, 1996). N_CCAP_ are among the best characterized peptidergic neurons in *Drosophila* both in terms of function (Park et al., 2003; Clark et al., 2004; Peabody et al., 2009, 2008; Lahr et al., 2012) and general morphology (Ewer and Truman, 1996; Park et al., 2003; Santos et al., 2007; Zhao et al., 2008; Veverytsa and Allan, 2012). Our previous work using the presynaptic marker syb::GFP and the postsynaptic marker rdl::HA (Sánchez-Soriano et al., 2005) and peptide immunostaining had suggested that both mixed in- and output as well as “pure” output compartments may exist for these neurons (Santos et al., 2007). We now test and elaborate on these findings by using additional markers and a combination of microscopical techniques.

Our results show that a small set of chemically similar peptidergic insect interneurons can have a remarkable variability regarding the spatial separation of in- and output compartments. Peptide release can take place both close and far away from synaptic sites, and both in- and output synapses can occur along the same neurite. We also found reciprocal synapses between peptidergic interneurons, suggesting a specialization that may aid in rapid and synchronized strong peptide release from different peptidergic neurons expressing the same peptide(s).

## Materials and methods

### Fly lines

The following Gal4-UAS fly strains were used: w^*^; Ccap-Gal4 (Park et al., 2003, kind gift of John Ewer, Valparaiso, Chile), w^*^; P{w^+*mC*^ = UAS-nsyb::eGFP} (Estes et al., 2000, Bloomington Stock Center), w^*^; P{w^+*mC*^ = UAS-syt::eGFP} (Zhang et al., 2002, Bloomington Stock Center), w^1118^; P{w^+*mC*^ = UAS-DenMark} (Nicolaï et al., 2010, kind gift of Bassem Hassan, Leuven, Belgium), UAS-GFP::shal2 (Diao et al., 2010, kind gift of Susan Tsunoda, Fort Collins, CO, USA), and UAS-Dscam17.1::GFP (Wang et al., 2004, kind gift of Tzumin Lee, Worcester, MA), UAS-GCaMP1.6 (Reiff et al., 2005, kind gift of D. Reiff, Freiburg, Germany), UAS-mCD8::GFP (Lee and Luo, 1999, Bloomington Stock Center) and UAS-10xmyrGFP (Bloomington Stock Center). For single cell stainings, yw hsp70-flp;Sp/CyO;UAS-CD2y^+^-mCD8::GFP/TM6b and yw hsp70-flp;UAS-CD2y^+^-mCD8::GFP/CyO; Tm3/TM6b (Wong et al., 2002, kind gift of Gary Struhl) were used.

### Generation of transgenic flies

To generate UAS-*capa* flies, the cDNA clone GH28004 in pOT2 (Berkeley *Drosophila* Genome Project Gold Collection) was cut with *Bgl*II and *Xho*I and the resulting *capa* insert was cloned into pUAST. The final pUAST vector was full-length sequenced to exclude errors. Transgenic flies were generated by VANEDIS injection service (Oslo, Norway).

### Immunostainings and GFP-labeling

CNS from third instar larvae were dissected in standard fly saline or PBS, fixed for 45 min to 4 h in 4% paraformaldehyde in 0.1 M sodium phosphate buffered saline (PBS, pH 7.2) at 4°C, washed in PBS with 1% TritonX (PBT) and incubated for at least 24 h in PBT containing 10% normal goat serum in combination with rabbit polyclonal (anti-PRXa (Eckert et al., 2002, 1:5000), anti-CCAP (Dircksen and Keller, 1988, 1:1000), anti-GABA (Sigma-Aldrich, 1:800, Thum et al., 2011), a-GFP (Invitrogen, 1:1000) or mouse monoclonal antibodies (a-brp nc82 (1:100), anti-GFP (Invitrogen) 1:1000), as well as a-FasII 1D4 (1:75), and a-ChAT 4B1 (1:50) obtained from the Developmental Studies Hybridoma Bank under the auspices of the NICHD and maintained by the University of Iowa. Preparations were washed 5 times during a day with PBT and incubated for at least 24 h in PBT containing 10% normal goat serum with DyLight488-, DyLight649- or Cy3-conjugated AffiniPure goat anti-mouse or goat anti-rabbit IgG (H+L; Jackson ImmunoResearch, Germany), used at a dilution of 1:1000. Preparations were subsequently washed for about 4 h, then mounted in 80% glycerol diluted in PBS. To avoid compression of the preparations, small plastic spacers were placed between the slide and cover glass. The nomenclature follows Selcho et al. (2009) for the larval brain areas, and Landgraf et al. (2003) for the FasII tracts in the thoracic and abdominal neuromeres. The nomenclature of Landgraf et al. was also applied for the FasII tracts in the brain and suboesophageal neuromeres originally described with a slightly different nomenclature by Nassif et al. (2003).

For flp-out single cell labeling (Wong et al., 2002; Selcho et al., 2009), Ccap-Gal4 flies were crossed to either yw hsp70flp;Sp/CyO;UAS>CD2y+>mCD8::GFP/ or yw hsp70flp; UAS>CD2y+>mCD8::GFP;TM2/TM6b stocks. Flies were incubated at 25°C, eggs were collected every morning and evening. After 60 h, the emerged L2 larvae were heatshocked at 37°C for 20 min. Then, larvae were put back to 25°C until L3 wandering larvae emerged. Out of around 1500 dissected heat-shocked brains, 225 showed a suitably restricted GFP-expression to analyse the morphology of single neurons, even though preparations with single cell expression were rare. For each neuron class, single cell expression could be obtained, but not for each cell in each neuromere. Based on the high degree of serial homology of N_CCAP_ in the ventral ganglion, the complete anatomical pattern described was achieved by a synthesis of single cell stainings and the more abundant preparations showing 2–10 neurons. For each cell type in a tagma, at least 4 different preparations allowing to trace the whole single cell morphology were analysed.

### Confocal microscopy and data analysis

Confocal stacks were acquired on a confocal laser scanning microscope [Germany: Leica TCS SP5 or SPE (Leica Microsystems, Wetzlar, Germany) with a 20× objective (ACS APO 20× N.A. 0.6 IMM), 40× objective (ACS APO 40× N.A. 1.15 oil) or 63× objective (ACS APO 63× N.A. 1.3 oil); Hungary: Olympus Fluoview FV1000 (Olympus MicroImaging, Japan) with a 20× objective (UPlanSapo N.A. 0.75) or 60× objective (UPlanSapo N.A. 1.35 oil)] at 512 × 512, 1024 × 512 or 1024 × 1024 pixel resolution in 0.5–1 μm steps along the z-axis.

Pictures were analysed using Leica LAS AF lite, version 2.4.1 and the Fiji image processing package (Schindelin et al., 2012). For 3D volume-rendering, image stacks were imported into AMIRA 5.3 software (Indeed-Visual Concepts, Berlin, Germany) and processed using the *Voltex*, *ObliqueSlice*, and *SkeletonTree* tool in Amira 5.3 (Schmitt et al., 2004; Evers et al., 2005). A false color map was applied to the volume-rendered neurons and brightness and contrast were adjusted. Snapshots were taken in AMIRA and processed with CorelDraw X6 (Corel Corporation, Ontario, Canada). Figures were generated with the help of Adobe Photoshop CS6 (Adobe Systems Inc.) using brightness and contrast adjustments.

### Immuno-electron microscopy

A series of central nervous systems of selected L3 state *Drosophila melanogaster* larvae were carefully and quickly dissected in cold Drosophila Ringer solution, freshly completed with glucose (128 mM NaCl, 5 mM KCl, 2 mM CaCl_2_, 10 mM glucose). Ringer solution was oxygenated for 30 min and adjusted to pH 7. Specimens were then transferred to Ringer solution containing 0.4 % tannic acid and incubated at 25°C for 30 min. In preliminary experiments comparing several parallel protocols with different buffers, concentrations and temperatures, parameters were optimized with focus on a satisfactory tissue preservation as well as preserved immunoreactivity. After rinsing in clear Ringer solution devoid of glucose, samples were transferred into 0.1 M phosphate buffered saline (PBS, pH 7.4), then fixed in a mixture of 0.5% glutaraldehyde and 4% paraformaldehyde for 3 h at room temperature.

Another series of specimens were dissected in ice-cold PBS and fixed in a mixture of 1% glutaraldehyde and 4% paraformaldehyde, omitting the tannic acid pre-fixation step.

Samples of both series were finally washed in PBS, post-fixed in osmium tetraoxide (1% in 0.1 M phosphate buffer; Sigma), dehydrated and embedded into Durcupan epoxy resin (Sigma). Serial ultrathin sections were cut in the sagittal and transversal plane of the ventral ganglion and collected on nickel grids. Following routine etching, de-osmication and several thorough rinsing in Tris- buffered saline complemented with 20 mM glycine, (TBS-Gly, pH 7.6), samples were pre-incubated on drops of 5% normal goat serum (NGS) in TBS for 30 min. Grids were then transferred onto drops of the primary anti serum, anti-PRXamide (raised in rabbit, diluted 1: 1000 in TBS; Eckert et al., 2002) for 2 h. Thereafter, grids were thoroughly washed in TBS several times and treated again with drops of 1% normal goat serum. Anti-rabbit IgG conjugated with 18 nm colloidal gold, diluted 1:30 in TBS (Jackson Immunoresearch) was used as a secondary antiserum for 2 h.

Controls for immunoreaction of the secondary colloidal gold-coupled antiserum were carried out by omitting the primary antiserum. Following application of the secondary antiserum, no attached colloidal gold grains were seen in the samples. At last, grids were rinsed in several drops of distilled water and counterstained routinely with uranyl acetate and lead citrate. Samples were observed and documented with a JEOL 1200 transmission electron microscope.

### Calcium imaging

For *in situ* imaging, whole larval CNS were dissected in hemolymph-like HL3 saline (HL3; Stewart et al., 1994) containing (in mM): 80 NaCl, 5 KCl, MgCl2, 1.5 CaCl2, 10 NaHCO3, 75 Sucrose, 5 trehalose, and 5 HEPES, pH 7.2. If not stated otherwise, the CNS was incubated for 3 min at room temperature in 1 ml HL3 containing 1 mg collagenase (Sigma) and 0.5 mg dispase (Gibco/Invitrogen) to increase tissue penetration, followed by 2× washing. The CNS was then transferred to a small drop of HL3 on a cover glass which was mounted in an imaging chamber as described by Vömel and Wegener (2007). Saline was removed and the tissue was fixed to the cover glass with 2% low melting agarose (AppliChem, Darmstadt, Germany) in HL3 warmed to 36°C. Immediately after hardening of the agarose, the imaging chamber was filled with HL3 and imaging was started. Drugs were dissolved in 1 ml HL3 and bath-applied. Washes were done with 3 ml HL3. Excess solution was removed by a cassette pump. Tetrodotoxin (TTX) was obtained from Alexis (Farmingdale NY, USA), carbachol was obtained from Sigma (Deisenhofen, Germany), and ETH-1 (DDSSPGFFLKITKNVPRLa) was synthesized via FMOC methodology according to previously described procedures (Nachman et al., 2009). In the TTX experiments, preparations were incubated in 10 μM TTX for 30 min prior to the application of carbachol or ETH-1 in HL3 containing 10 μM TTX.

The imaging system consisted of an Axiovert 200 microscope (Zeiss, Jena, Germany) equipped with a Zeiss 40× Fluar oil immersion objective (NA 1.3), a conventional FITC filter set (Chroma, Brattleboro, VT), and a cooled CCD camera (Hamamatsu C4742-80-12AG, Hamamatsu Photonics, Herrsching, Germany). Excitation light at 483 nm was provided by a Polychrome IV system (T.I.L.L. Photonics, Gräfelfing, Germany) equipped with a computer-controlled shutter. Light was attenuated by a quartz neutral density filters (50%) to prevent photo-damage of the cells. OpenLab 4.0 software (Improvision, Warwick, UK) on an Apple Macintosh G5 PowerPC was used for system control and image acquisition. Images were typically acquired with an intensity resolution of 12 bit at 0.3–0.5 Hz after background subtraction, with 4 × 4 binning resulting in a pixel resolution of 336 × 256. Baseline subtraction and plotting was performed with OriginPro 9G (OriginLab Corporation, Northampton, MA, USA).

## Results

**Figure 1:**
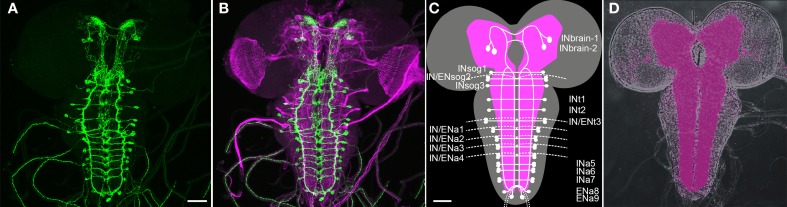
**Overview of the distribution of CCAP neurons (N_CCAP_) in the CNS of a 3rd instar larva (maximum projections). (**A** + **B**)** Immunostaining against mCD8::GFP ectopically driven by Ccap-GAL4 (green) merged in **(B)** with a staining against FasII and choline acetyltransferase (ChAT) to visualize tracts and neuropiles (magenta). **(C)** Scheme of the distribution of N_CCAP_ somata and main projections, showing the nomenclature of identified cells used in this work: IN/EN signifies interneurons and efferent neurons respectively. The lower script indicates the respective tagmata: brain, suboesophageal (sog), thoracic (t) and abdominal (a) neuromeres. There are two IN pairs in the brain, and one IN pair in each of the ventral ganglion neuromeres except a8/9. In addition, a pair of EN is found in sog2, t3, and a1–4, and a8/9. The scheme is based on the nervous system in **(D)**, showing the neuropile stained against the synaptic protein bruchpilot (nc82 antiserum). Scale bars = 50 μm.

Previous work has shown that Ccap-Gal4 is expressed in 46 neurons (N_CCAP_) throughout the larval CNS: 2 pairs with somata in the brain, 1 pair with somata in the suboesophageal neuromeres (sog) sog1 and −3, 1 pair in thoracic neuromere t1-t2 and abdominal neuromere a5–9 and 2 pairs in sog2, t3 and a1–4 (Figure 1, Ewer and Truman, 1996; Park et al., 2003; Santos et al., 2007; Zhao et al., 2008). In t3-a4 -the ventral ganglion neuromeres with two pairs of N_CCAP_- one neurite on each site leaves the central neuropile to exit via the respective segmental nerve (Santos et al., 2007; Zhao et al., 2008). These neurites form type III neurohaemal terminals on the body wall muscles M12 and M13 (Hodge et al., 2005; Vömel and Wegener, 2007; Zhao et al., 2008). Two additional efferent neurites exit through each hindgut nerve of the posterior-most abdominal neuromeres; their termination is unknown (Zhao et al., 2008). During pupariation, further “late N_CCAP_” differentiate and become CCAP- and bursicon-immunopositive: an additional pair in neuromeres a5–a7 and a9 (Veverytsa and Allan, 2012), bringing the neuron number up to 54 prior to the time of pupation.

The projection patterns of N_CCAP_ in the larval thoracic and abdominal neuromeres have been described in detail by Santos et al. (2007) with relation to the FasII landmark system (Landgraf et al., 2003). Prominent features of the N_CCAP_ are fibres following the FasII-positive ventrolateral (VL) and dorsomedial (DM) tracts, and dense arborizations between the DM and ventromedial (VM) tracts. The description relating to the FasII tracts below is based on this data.

In the larva, all N_CCAP_ express the neuropeptide CCAP (Park et al., 2003; Santos et al., 2007). The N_CCAP_ in a1–4 and in a8/9 further co-localize myoinhibitory peptides (MIPs, Kim et al., 2006; Vömel and Wegener, 2007), while the N_CCAP_ in the ventral ganglion co-localize the large neuropeptide bursicon (Dewey et al., 2004; Zhao et al., 2008).

From the previous anatomical descriptions of CCAP neurons, it remained unclear (i) which of the N_CCAP_ in t3-a4 projects to body wall muscle, (ii) which somata in the ventral ganglion give rise to the projections along the FasII-positive VL and DM tracts, and (iii) which somata give rise to the median arborizations around the DM tracts. Since knowledge of the full anatomy of single N_CCAP_ is crucial to identify in- and output regions of the N_CCAP_, we first characterized their projections on the single-cell level by the flp-out technique (Wong et al., 2002). We next analysed the distribution of peptide immunoreactivity, and vesicle/dendritic markers genetically expressed by the GAL4/UAS-system (Brand and Perrimon, 1993). To test whether the resulting marker distribution correlates with synaptic events in selected structures, we used immunoelectron microscopy (immuno-EM). Since the commonly used CCAP antiserum (Dircksen and Keller, 1988) did not work well in immuno-EM, and since MIP and bursicon only occur in subsets of the N_CCAP_ (see above), we ectopically co-expressed the *Drosophila capa*-gene by Ccap-Gal4 in many preparations. The CAPA prepropeptide codes for three peptides ending on the C-terminal sequence PRXamide (Kean et al., 2002) specifically recognized by an anti-PRXa serum (Eckert et al., 2002) that has worked reliably with high specificity in immuno-EM in cockroaches (Pollák et al., 2005) and fruitflies (Santos et al., 2006). All immunolabeling on the EM level reported below is due to anti-PRXa staining of ectopically expressed CAPA peptides. Native expression of CAPA peptides is restricted to 8 neurons in the larval ventral ganglion (Kean et al., 2002; Santos et al., 2006, 2007) with ventral ganglion projection patterns that could easily be separated from the N_CCAP_ as pretested by 3D reconstructions.

### Singe cell morphology of the different CCAP neuron classes

#### N_CCAP_ in the brain

**Figure 2:**
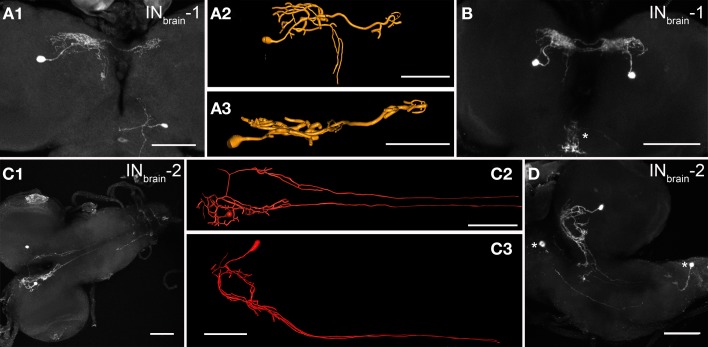
**Morphology of the two pairs of N_CCAP_ interneurons (IN_brain_-1/-2) in the brain of 3rd instar larvae. (A1)** The IN_brain_-1 shows a large-field projection in the ipsilateral dmp and dlp, and a smaller arborizations in the contralateral dmp (maximum projection). The same neuron was reconstructed and is shown from dorsal **(A2)** and posterior **(A3)**. **(B)** The pair of IN_brain_-1 neurons (maximum projection). The arborizations on the ipsi- and contralateral sides are overlapping. The arborizations in the sog (asterisk) are from a different neuron with soma in the sog. **(C1)** The IN_brain_-2 arborizes in the ipsilateral dmp, and sends bilateral descending neurites along the DM tracts which are obscured in their posterior part by the maximum projection, but are visible in the reconstructions in **(C2)** (dorsal view) and **(C3)** (lateral view). The circular structure formed by the IN_brain_-2 neurites is only visible in the lateral view as shown in another preparation in **(D)**, which shows additional N_CCAP_ in the ventral ganglion (asterisks). Scale bars = 50 μm.

In each brain hemisphere, one interneuron (IN_brain_-1) located in the posterior basomedial protocerebrum (bmp) branched first in the ipsilateral dorsomedial protocerebrum (dmp) with some extensions reaching the dorsolateral protocerebrum (dlp), then within a more confined area in the contralateral dmp (Figures 2A,B). In most cases, a neurite emanated from the ipsilateral branchings that descends posteriorly through the bmp along the foramen and arborizes at variable positions within the bmp before reaching the suboesophageal neuromeres. The inversely arranged arborization pattern of the paired IN_brain_-1 lead to overlapping arborizations in the dmp (Figure 2B). The ipsilateral arborizations in the dmp and dlp had a “smooth” appearance and showed only very small overlap with peptide immunoreactivity (see Figure 8A below). In contrast, the contralateral arborizations in the dmp showed varicosities with co-localized CAPA immunoreactivity. Based on morphological criteria, the ipsilateral branches may therefore represent a dendritic compartment, while the contralateral arborizations may represent an output compartment from which peptides are released.

Also the second pair of brain interneurons (IN_brain_-2) was bilaterally symmetric, with somata located in the posterior bmp. The primary neurite of IN_brain_-2 branched very medially in the dmp, in a region partially overlapping with branches of the IN_brain_-1 neurons, and projected along the foramen forming shorter arborizations in the bmp. These arborizations formed a ring-like structure (Figures 2C,D). One neurite from the dorsomedial protocerebrum crossed the midline dorsal to the foramen and projected contralaterally. It then descended along the foramen analogue to the neurite on the ipsilateral side, but without extensive branching (Figure 2C). After having left the brain, the descending neurites on both sides followed the DM tract (Santos et al., 2007) and ended blindly at a variable position in abdominal neuromer a4–7. Thus, the projections of each IN_brain_-2 form an H-like morphology, and it is this neuron type that gives rise to the descending N_CCAP_ neurites along the DM tract.

A pair of weakly stained neurons in the anterior bmp were also visible in most preparations, especially when expressing UAS-10x-myrGFP. The neurites of these cells could not be immunolabeled against CCAP, nor did the cells occur in our single cell labeling. We therefore think that these cells represent a weak and CCAP-unspecific expression of Gal4 that became visible. Few cells with similar features were occasionally also found in the ventral ganglion (VG).

#### N_CCAP_ in the suboesophageal neuromeres

**Figure 3:**
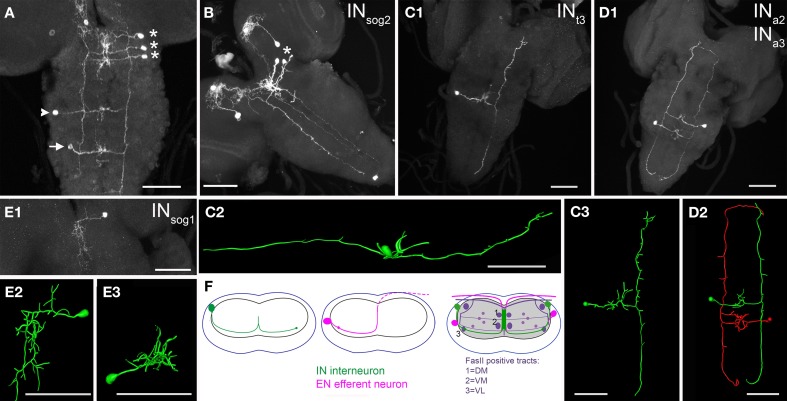
**Morphology of N_CCAP_ interneurons (IN) in the ventral ganglion of 3rd instar larvae. (A)** One IN in each suboesophageal neuromere (asterisks) on the right side, plus one thoracic IN (arrowhead) and abdominal IN (arrow) on the left (contralateral) side is labeled (maximum projection). Each cell shows arborizations around the midline, and projects to the contralateral side where the neurite splits to project both anterior and posterior along the VL tract. Thus, the labeling of the VL tract of the left side originates from the IN_sog_, while the labeling of the VL tract of the right side originates from the other INs. **(B)** While several N_CCAP_ are labeled in this preparation, the pattern contains only one IN in the ventral ganglion (asterisk). The projection of this IN_sog2_ along the VL tract spans the entire length of the ventral ganglion (maximum projection, ventral view). **(C1)** Single cell morphology of an IN_t3_. Also here and in the reconstructions **(C2)** (lateral view) and **(C3)** (dorsal view), the median arborizations, and the IN-typical projections along the VL tract through the entire length of the ventral ganglion is visible (maximum projection, ventral view). **(D1)** Maximum projection of one IN_a2_ and one contralateral IN_a3_ (dorsal view). The pair is reconstructed in ventral view in **(D2)**, highlighting the typical morphology of an IN in the ventral ganglion. **(E)** IN_sog1_ are the only IN in the ventral ganglion that lack the contralateral projection and neurites along the VL tract. Typical for an IN_sog_, the arborizations around the midline are more extended in the anterior-posterior axis than in the abdominal neuromeres, and reach also to the other sog neuromeres. **(E1)**: maximum projection, **(E2)**: reconstruction, ventral view, **(E3)**: reconstruction, anterior view). **(F)** Generalized scheme of the projection patterns of IN and EN in the ventral ganglion. The dot represents the VL tract, where the EN fibres show small arborizations on the ipsilateral side, and the IN project along on the contralateral side. The dashed line in the middle scheme marks the part of the efferent neurite outside of the neuropile but still within the ganglion. Scale bars = 50 μm.

A bilaterally symmetric interneuron pair (IN_sog1 − 3_) was located in each suboesophageal neuromere (sog1–3, Figures 3A,B). The primary neurite of each neuron projected medially, and showed extensive arborizations along the midline between DM and VM fascicles of the homotopic and the neighboring sog segments (Figure 3E). Smaller arborizations were located very close to the cell body, substantially lateral to the VL fascicle. In IN_sog2 − 3_, the primary neurite crossed the midline, and continued ventrally into the contralateral neuropile to branch in a T-shape at the VL tract. The long posterior branch followed the VL tract until its posterior end, then the neurite bent medially. The shorter anterior branch also followed the VL track until the anterior end at the sog-brain border, then it bent medially forming small arborizations (Figures 3A,B).

**Figure 4:**
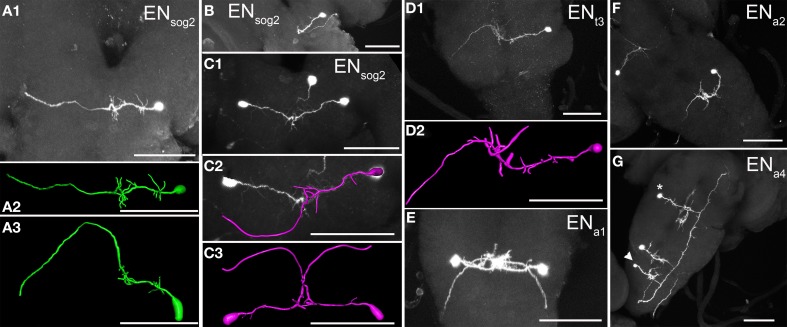
**Morphology of efferent N_CCAP_ (EN) in the ventral ganglion of 3rd instar larvae. (A1)** The sog only contains one pair of EN in neuromere 2, which leaves the ventral ganglion via the maxillary nerve (maximum projection) on the contralateral side. A reconstruction of this neuron is shown from ventral **(A2)** and posterior **(A3)**. **(B)** In a few single cell preparations, ENs projected to the maxillary nerve of the ipsilateral side (maximum projection). It is unclear whether this represents an alternative projection pattern of EN_sog2_. **(C1)** A pair of EN_sog2_ neurons from ventral (maximum projection). The same neurons reconstructed in dorsal view **(C2)** and posterior view **(C3)** assuming a contralateral projection. Due to the overlap of neurites in the midline, it was only possible to reliably identify neurite identity in single cell preparations. **(D1)** The efferent neuron in thoracic neuromere 3 (maximum projection) sends its neurite to the contralateral side where it leaves the ventral ganglion via the segmental nerve. As in all other ENs, the dorsal U-shaped projection along the dorsal neuropile edge is visible, especially in the slightly angled dorsal view of the reconstruction in **(D2)**. **(E)** Dorsal view of a pair of efferent neurons in abdominal segment a1 (maximum projection). The EN in the abdominal neuromeres show a stereotypic morphology well visible in the single cell in a2 in **(F)**. The somata are located in a more dorsal position than in the sog. Thus the neurites project first ventrally, then bend dorsally at the midline, and then form the characteristical dorsal U-turn on the contralateral side to leave the ventral ganglion via the segmental nerve. **(G)** An EN in abdominal segment a4 (maximum projection), together with an interneuron (IN) in a thoracic neuromere (asterisk) and in a5 (arrowhead). Scale bars = 50 μm.

Veverytsa and Allan (2012) classified all N_CCAP_ in the sog as INs, yet it is clear from our data that sog2 contains one pair of efferent neurons. These EN_sog2_ projected a primary neurite medially, which showed arborizations close to the cell body and between the ipsilateral DM and VM fibre of the homotopic segment. In most preparations (Figures 4A,C), the neurite continued to the contralateral side where it projected dorsally and then ventrally, forming a turn that appeared to follow the dorsal border of the contralateral neuropile. The neurite then left the sog2 via the contralateral maxillary nerve. In a few preparations, however, the neurite formed a similar trajectory but remained entirely on the ipsilateral side (Figure 4B).

#### N_CCAP_ in the thoracic neuromeres and abdominal neuromeres 1–7

While thoracic neuromeres t1–2 contained only one bilaterally symmetric pair of N_CCAP_ interneurons (INt_1−2_), t3 held 1 pair of ventrally located interneurons (INt_3_) plus one pair of efferent neurons (EN_t3_) (Figure 1). The EN_t3_ and IN_t3_ somata on each side typically lay closely together, approximately at the height of the VL tract. While EN_t3_ was typically more laterally situated than IN_t3_, either soma was less than a cell-diameter more dorsal or ventral than the other. Like in t3, a bilateral pair of IN and EN were found in a1–4 (Figure 1). Their somata located to a more dorsal position, between the height of the VL and the DL tract. The IN and EN somata in each hemineuromere a1–a4 located often closely together at variable positions, but the EN tended to occupy a more ventrolateral position (Figure 3F) than the IN. In a5–7, typically only one (very rarely two) pair of lateral interneurons (IN_a5−7_) could be marked (Figure 1). Their somata also resided between the VL and DL tract in the dorso-ventral axis.

Each N_CCAP_ in t1–3 and a1–7 sent a primary neurite ventromedially until the midline (Figures 3C,D, 4D–G). The IN neurites then strongly arborized dorsally between the DM and VM fascicles. These medial arborizations extended to the segment borders. The primary neurite then projected ventrolateraly into the contralateral neuropile and branched in a T-shape at the VL fibre (Figures 3C,D). Independent of the neuromer of origin, the T-shaped branches projected through the whole or a large part of the ventral ganglion as described for the IN_sog_ projections (Figures 3C,D). Thus, the CCAP-positive fibres along the VL tract on each side appeared to be composed of up to 12 individual N_CCAP_ neurites at least in the middle portion of the ventral ganglion. In some preparation the VL fibre bent within the terminal plexus and projected a short distance anterior along the VM tract, reaching a7/a8. Since this projection was rarely seen in single cell preparations, we are unable to state whether all or only the posteriormost INs of the ventral ganglion contribute to this bend.

The EN neurite also gave off branches between the VM and DM tract, but then projected mediodorsally, and then dorsolaterally to innervate the contralateral segmental nerve (Figures 4D–G).

#### N_CCAP_ in the abdominal neuromeres 8/9 of the ventral ganglion

**Figure 5:**
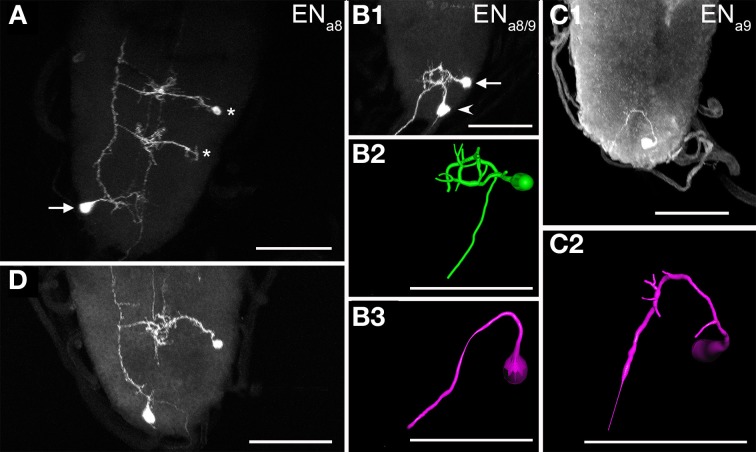
**Morphology of efferent N_CCAP_ (EN) in abdominal neuromeres a8/9 of 3rd instar larvae. (A)** The EN_a8_ (arrow) show relatively sparse arborizations in the terminal plexus, and project through the contralateral segmental nerve 8 to the periphery. Two INs are also visible (asterisks), with projections along the VL fibre (compare Figure 4). **(B1)** Maximum projection of one EN_a8_ (arrow) and one EN_a9_ (arrowhead) of the same side (dorsal view), and respective reconstructions **(B2,B3)**. Unlike in A) EN_a8_ appears to project via the ipsilateral segmental nerve 8 in this preparation. EN_a9_ shows no discernible arborizations and projects via the contralateral segmental nerve 8. **(C1)** Maximum projection of a preparation with a single marked EN_a9_ neuron, reconstructed in **(C2)**. This neuron shows small arborizations in the terminal plexus, and projects via the contralateral segmental nerve 8 to the periphery. Scale bars = 50 μm.

Segments a8 and a9 each contained a bilaterally symmetric pair of N_CCAP_ with efferent projections through the hindgut nerve (containing the fused segmental nerves 8 and 9, Figure 5). One pair (EN_a8_) was located ventrolaterally (Figures 5A,B), anterior to the other pair (EN_a9_) which was more medially located, close to the posterior tip of the ventral ganglion (Figures 5B–D). Each EN_a8_ sent a primary neurite medially toward the midline (Figures 5A,B). Typically, the primary neurite split into two branches with small arborizations in the ipsilateral neuropile. Denser arborizations were seen around the DM fibres similar to the situation of the EN_a1−4_. The primary neurite of EN_a8_ then projected posteriorly and typically innervated the contralateral hindgut nerve (Figure 5A, but see also Figure 5B). EN_a9_ showed a comparable projection pattern to EN_a8_, but arborized at best only very little around the DM fascicles. Since EN_a9_ somata were located at the posterior tip, their primary neurites projected first anteriorly to the same location around the DM fibre as the EN_a8_, and then innervated the contralateral hindgut nerve (Figures 5B–D).

**Figure 6:**
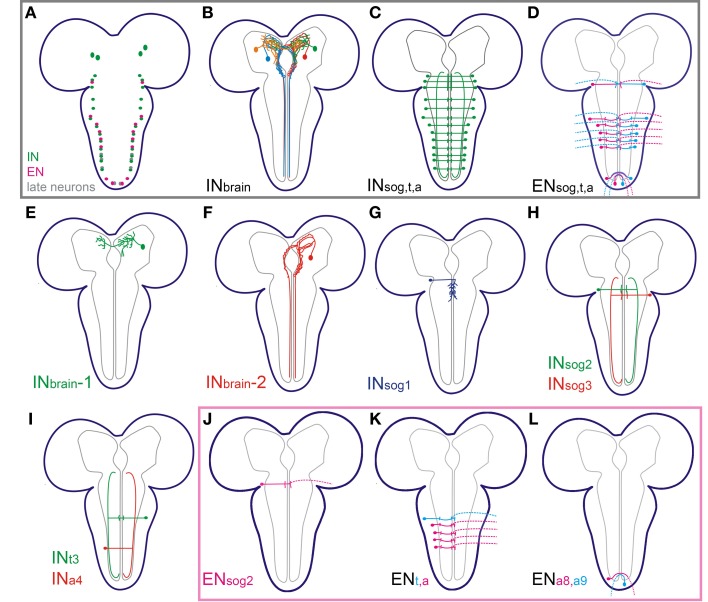
**Summary of the morphology of the different N_CCAP_ in 3rd instar larvae. (A)** Somata distribution of interneurons (IN, in green: two pairs in the brain; one pair in all ventral ganglion neuromeres but a8/9), efferent neurons (EN, in magenta: one pair in sog2, t3, a1–4 and a8/9) and late neurons (in gray) that appear after pupariation (Veverytsa and Allan, 2012). The late neurons were not observed in the Ccap-GAL4 expression pattern in 3rd instar larvaed and are not described in this work. **(B)** Summary of the major projections of the IN in the brain, and ventral nerve cord **(C)**. **(D)** Summary of the major projections of the EN. **(E–I)** Single cell morphology of different INs as indicated. **(J–L)** Single cell morphology of different ENs as indicated.

The single cell anatomy for the N_CCAP_ is schematically summarized in Figure 6. Based on our results, the N_CCAP_ (Figures 6A–D) can be classified into five morphologically distinct groups: (1) local interneurons in the protocerebrum (IN_brain_-1, Figures 6B,E), (2) projection neurons with somata in the protocerebrum and descending projections through the ventral ganglion along the DM tract (IN_brain_-2, Figures 6B,F), (3) projection neurons in the ventral ganglion with long neurites along the VL tract (IN_sog2−3_, IN_t_, IN_a_, Figures 6C,H,I), (4) local interneurons with medial branches throughout the sog (IN_sog1_, Figure 6G), and (5) efferent neurons with neurohaemal release sites in the periphery (EN_sog2_, EN_t3_, EN_a1−4_ and EN_a8−9_, Figures 6D,J–L).

### Assignment of in- and output sites by fluorescent vesicle markers

#### Fluorescent dendritic markers

To identify putative dendritic input sites of the N_CCAP_, we ectopically drove the expression of three different GFP-tagged dendrite markers GFP::shal2 (Diao et al., 2010), UAS-DenMark (Nicolaï et al., 2010), UAS-Dscam17.1::GFP (Wang et al., 2004) in the N_CCAP_ together with UAS-*capa.* The CNS of resulting wandering L3 larvae were then stained against PRXamide and—if appropriate—against GFP.

**Figure 7:**
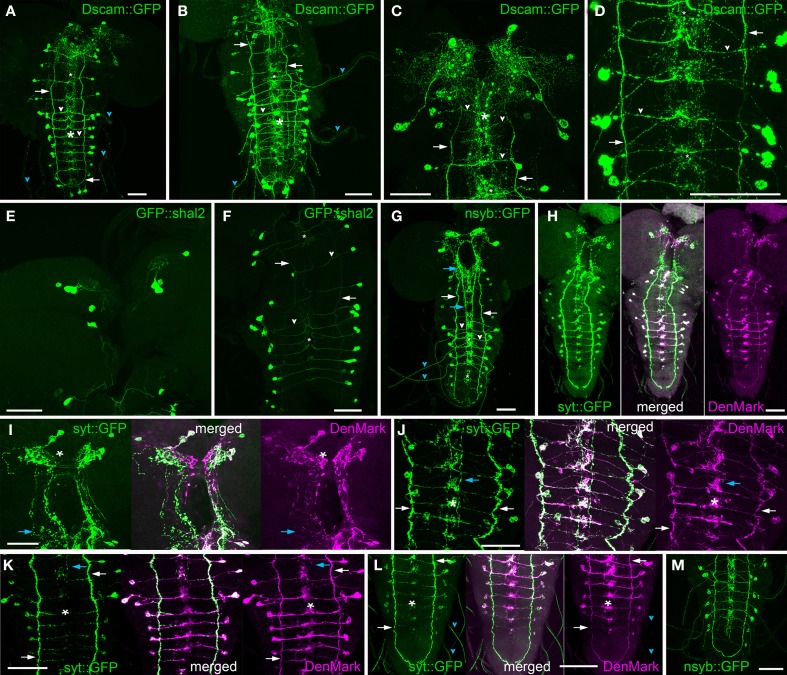
**Distribution of fluorescent vesicle and dendritic markers in 3rd instar larvae. (A–D)** Maximum projection of nervous systems of flies expressing the dendrite marker Dscam17.1::GFP in N_CCAP_ (anti-GFP staining). **(A)** CNS overview. **(B)** Ventral ganglion overview. Details of the brain **(C)** and abdominal neuromeres **(D)**. Besides the somata, the VL fibres (arrows), transverse projections (white arrow heads), segmental nerves (cyan arrow heads) and median arborizations (asterisks) in the ventral ganglion and a relative broad neuropile area in the dorsomedian protocerebrum **(C)** are strongly labeled. The labeling is distributed in small punctae that become partly fused along the VL fibre and transversal projections. **(E,F)** Maximum projection of nervous systems of flies expressing the dendrite marker GFP::shal2 in N_CCAP_ (anti-GFP staining) in the brain **(E)** and ventral ganglion **(F)**. Though the somata are strongly labeled, only few arborizations and projections including the transverse projections (arrow heads) are strongly labeled. The VL fibres (arrows) and median arborizations (asterisks) in the ventral ganglion are less strongly labeled. **(G)** CNS maximum projection of a larval CNS expressing the vesicle marker nsyb::GFP in N_CCAP_ (anti-GFP staining). As for the dendrite markers, somata, VL fibres (white arrows) and segmental nerves (cyan arrowhead) are labeled. In contrast, the descending neurites of the IN_brain_-2 are strongly marked around the foramen and along the DM tract in the ventral ganglion (cyan arrows), while the median arborizations (asterisks) are relatively weakly labeled in a more restricted area. Only the transverse projections (white arrowhead) of the EN in the ventral ganglion are stained. **(H–L)** Maximum projection of larval nervous CNS co-expressing the vesicle marker syt::GFP (green, anti-GFP staining) and the dendrite marker DenMark (magenta, RFP fluorescence) in N_CCAP_. (**H)** overview, ventral view. **(I)** brain, **(J + K)** thoracic and abdominal neuromeres of a preparation with strong **(J)** and rather weak **(K)** syt::GFP expression. From the merged projections, it is visible that syt::GFP and DenMark labeling is not entirely overlapping. In the median protocerebrum **(I)**, the most median part is exclusively DenMark labeled. DenMark labeling, albeit weak, occupies a larger area than syt::GFP in the dorsolateral protocerebrum (asterisk in **I**). Also the area of labeling of the arborizations between the DM and VM fascicles (asterisks) in the ventral ganglion is clearly larger for DenMark than for syt::GFP (**J** and **K**). In contrast, the descending fibres of the IN_brain_-2 (cyan arrows) are only marked by syt::GFP, DenMark labeling is completely absent. Also the segmental nerves are strongly labeled by syt::GFP, but only faintly marked by DenMark (cyan arrowhead). In contrast, the fibres in the VL tract (white arrow) are strongly labeled by both markers in all preparations. **(L)** abdominal neuromeres. **(M)** Maximum projection of the thoracic and abdominal neuromeres CNS of a larva expressing the vesicle marker nsyb::GFP in N_CCAP_ (anti-GFP staining). Comparing syb::GFP labeling in **(M)** and **(G)** with syt:GFP in **H–L**), it is visible that the descending IN_brain_-2 neurites (cyan arrows) are more strongly stained by syb::GFP. Scale bars = 50 μm.

**Figure 8:**
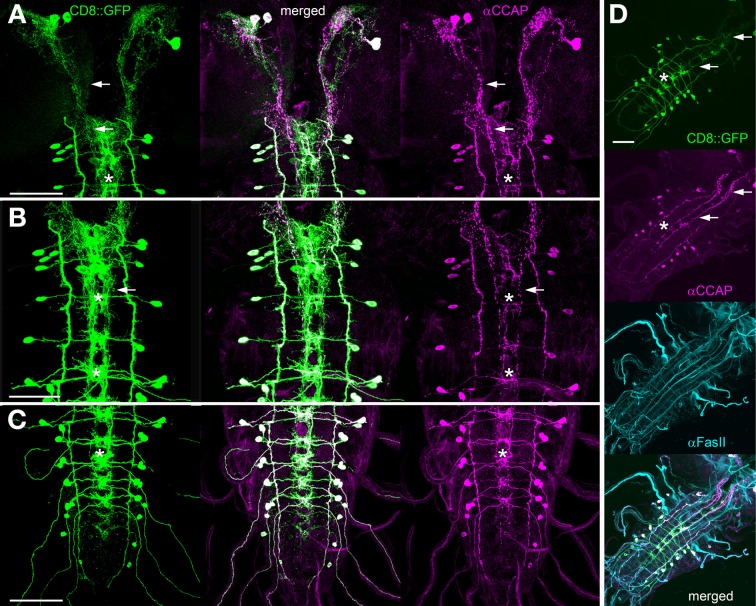
**Comparison of Ccap-GAL4-driven expression of mCD8::GFP (green) and CCAP immunoreactivity (magenta, maximum projections). (A)** Brain and sog, **(B)** sog and thoracic neuromeres, **(C)** t3 and abdominal neuromeres. **(D)** Overview of a CNS co-stained against FasII (cyan). While mCD8::GFP is expected to label the whole neurons, the descending IN_brain_-2 neurites are nearly exclusively marked by CCAP immunoreactivity in the area ventrolateral of the foramen (arrow in **A** and **D**). In contrast, CCAP staining is very restricted in the median ventral ganglion arborizations (asterisks), which are distinctly marked by mCD8::GFP. In **(C)**, the stronger impact of neural sheath background signal in the thin segmental nerves leads to the impression of a weaker axonal CCAP staining in the segmental nerves. Scale bars = 50 μm.

The overall distribution of all three dendritic markers was similar (Figures 7A–F, H–L). Besides the cell bodies, the following structures were labeled: arborization of IN_brain_ neurons in the dorsal protocerebrum, arborizations around the DM/VM tract in the ventral ganglion, the VL fibres, the terminal plexus and the primary neurites projecting perpendicular to the midline within the ventral ganglion neuropile. In contrast to the vesicle markers described below, DenMark and Dscam17.1::GFP more strongly and extensively labeled the arborizations around the DM/VM tracts in the ventral ganglion, but consistently failed to label the descending fibres of the IN_brain−2_ neurons (Figures 7A–D,H–L) that were strongly CCAP- and CAPA-immunoreactive (Figure 8). GFP::shal2 gave the weakest labeling throughout, which was mostly restricted to primary and main neurites (Figures 7E,F). Thus, the arborizations in the brain and ventral ganglion were not marked by GFP::shal2. In a few preparations, strong DenMark-labeled granular bodies could be found which were not observed by any other marker. These preparations were excluded from our analysis as we cannot exclude that they represent artefacts.

#### Fluorescent vesicle markers

To identify putative output sites of the N_CCAP_, we ectopically drove the expression of two different GFP-tagged vesicle markers (UAS-nsyb::eGFP (Estes et al., 2000) and UAS-syt::eGFP (Zhang et al., 2002) in the N_CCAP_ together with UAS-*capa.* The CNS of resulting wandering L3 larvae was then stained against PRXamides and GFP.

The overall distribution pattern of syb::GFP and syt::GFP was similar (Figures 7G–M). The cell bodies showed punctate stainings, possibly localized to endoplasmatic reticulum and Golgi compartments. Also along the neurites, punctate varicose staining was typical. Strongly stained structures included the varicose arborizations and descending neurites of the IN_brain_ in brain and ventral ganglion as well as the projections of ventral ganglion neurons along the VL tract. The primary neurites of ventral ganglion neurons that projected toward/away from the midline were less strongly but more continuously (less punctated) stained. The arborizations of the ventral ganglion neurons around the DM/VM tracts were not or only weakly labeled by syt::GFP—in no case could the full arborization pattern as labeled by mCD8::GFP or 10xUAS-myrGFP be visualized. More pronounced labeling of the DM/VM arborizations were visible with syb::GFP especially in t3 and a1–4, though again the full arborization pattern was not visible. Both markers were also found in the terminal plexus (Figures 7G–M).

In all preparations, the varicose CCAP or CAPA staining colocalized consistently with the vesicle markers. This indicates that (i) the vesicle markers label peptidergic vesicles, and (ii) that ectopically expressed CAPA peptides are similarly distributed as the endogenous peptides CCAP, MIP and bursicon. In cockroaches, CCAP and bursicon have been found to be partially co-packaged in the same DCV (Woodruff et al., 2008). Inversely, the distribution of vesicle marker and peptide staining showed a good match for syt::GFP, but not consistently for syb::GFP. This likely is attributable to the presence of small synaptic vesicles (SSV) that can also be labeled by the vesicle markers yet do not contain peptides (see below). While peptide and syb::GFP staining matched perfectly in brain arborizations and along the VL tract, syb::GFP staining was often not matched by peptide staining in the arborizations around the VM/DM fibres in the ventral ganglion, as well as along the primary N_CCAP_ neurites projection perpendicular to the midline. These arborizations and primary neurites are nearly fully labeled by the vesicle markers, while only partly and in a punctuate fashion labeled by peptide-immunoreactivity. Especially in the arborizations around the VM/DM fibres, larger stretches could only be visualized by vesicle marker staining. This finding correlates well with CCAP immunostainings, which in the ventral ganglion strongly labels the VL fibres and the descending fibres of the IN_brain_-2 neurons, while CCAP-immunoreactivity is mostly absent from the arborization around the DM/VM tracts (Figure 8, see also Santos et al., 2007).

#### Co-expression of syt::GFP and Denmark

To refine differences in the distribution of synaptic and dendritic markers, we co-expressed DenMark and syt::GFP within the N_CCAP_ (Figures 7H–L). As expectable from the individual staining patterns, most structures could be labeled by both markers albeit to a different extent. Somata and the VL fibres were strongly labeled by both markers, while the projections of ventral ganglion neurons perpendicular to the midline were less intensely labeled by both markers. The arborizations around the DM/VM tract were, however, more strongly labeled by DenMark than by syt::GFP. Moreover, the extent of visualized arborizations was discernibly higher for DenMark than for syt:GFP. We also found that the strongly varicose projections of the IN_brain_-2 neurons in the posterior brain and sog were exclusively labeled by the vesicle markers. Several regions of the innervated brain neuropiles also showed exclusive labeling with either one of the markers. The ipsilateral smooth arborizations of the the IN_brain_-1 and -2 in the dmp are strongly labeled by DenMark but are mostly devoid of co-localizing syt::GFP. In contrast, the more restricted contralateral varicose arborizations of the IN_brain_-1 are consistently labeled by syt::GFP but cotained only occasionally a weak DenMark signal (Figure 7I). This supports the earlier notion based on single cell stainings that the ipsilateral arborizations of the IN_brain_ neurons represent dendritic compartments, while the contralateral arborizations of the IN_brain_-1 neurons represent an output site overlapping with the dendritic arborizations of the IN_brain_-1 neurons of that contralateral site.

**Figure 9:**
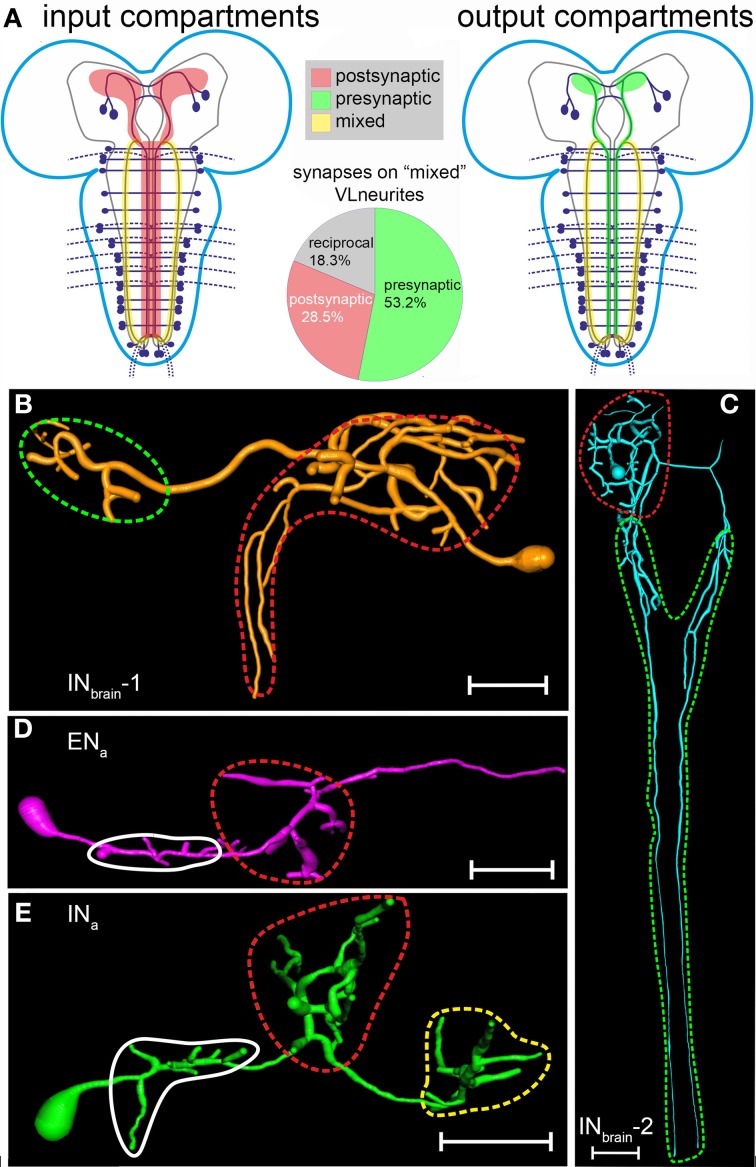
**Distribution of in- and output compartments (“dendritic/axonic segments”) on the N_CCAP_ based on the summarized results from ectopically expressed fluorescent markers and immuno-electron microscopy (for the IN_a_) or fluorescent markers alone (all other N_CCAP_). (A)** Left: major input compartments (labeled in red) of the N_CCAP_ network based on the distribution of the dendritic markers Dscam17.1::GFP and DenMark. These compartments consists of neurite divisions in the dorsomedial protocerebrum, the arborizations in the median ventral ganglion and the VL fibres. Right: major output compartments (labeled in green) of the N_CCAP_ network based on the distribution of the vesicle markers nsyb::GFP, syt::GFP and anti-CCAP immunostaining. These compartments consists of neurite divisions in the protocerebrum, the descending IN_brain_-2 neurites along the DM tract, arborizations in the median ventral ganglion, the VL fibres, and peripheral peptide neurohaemal release sites (not shown, see Vömel and Wegener, 2007). The VL neurites (shown as mixed in-and output compartment in yellow) are strongly stained by fluorescent dendritic and vesicle markers as well as the CCAP antiserum, suggesting the presence of both in- and output sites along the fibre. This view is fully supported by the frequency of the different synapse types observed by electron microscopy along the VL neurites (pie chart in the middle). **(B–E)**: In- and output compartments broken down to the single neuron types. **(B–D)** Input compartments are encircled in red. Output compartments presumably only releasing peptides are encircled in green. **(E)** In the INa and INt, the situation is more complex. The DM/VM arborizations encircled in red contain synaptic in- and output sites. The VL neurites encircled in yellow contain in- and output synapses plus peptide release sites. The small branches encircled in white could not be assigned, but are likely to represent dendritic compartments. Scale bars = 50 μm.

When combined, the results of single neuron morphology (Figure 6) and the distribution of vesicle/ dendritic markers (Figure 7) and CCAP/CAPA immunoreactivity (Figure 8) suggest the following general conclusions on N_CCAP_ polarity (Figure 9): (i) IN_brain_ neurons have separated input compartments (dendrites) and output compartments (peptide release sites, Figures 9B,C); (ii) The EN of the ventral ganglion likely have a mixed input- and (possibly non-peptidergic) output sites at the arborizations around the DM/VM tract (Figure 9D), (iii) the IN of the ventral ganglion have mixed synaptic input and output plus peptidergic output sites at the VL fibre and (iv) mixed synaptic input- output sites with no or very little peptide release at the arborizations around the DM/VM tract (Figures 9A,E); (v) the EN/IN neurites perpendicular to the midline in the ventral ganglion most likely do not represent in/output sites, but are labeled by transit vesicles containing markers or peptides (Figures 7, 8).

These conclusions are based on confocal light microscopy with limited spatial resolution and the untested assumption that the vesicle and dendritic markers specifically label in- and output sites in peptidergic neurons. To test these conclusions, we used immuno-electron microscopy as a high-resolution method to analyse in- and output synapses. We defined a synaptic event as a presynaptic structure showing dense bars and/or an associated agglomerate of synaptic vesicles together with a postsynaptic membrane thickening. To additionally reveal possible non-synaptic peptide release sites, we employed a combined aldehyde-tannic acid fixation protocol. Though aldehyde-tannic acid fixation does affect both fine structure and immunoreactivity, it allows to capture both events of dense core vesicle (DCV) exocytosis (Buma et al., 1984) and synaptic structures, and has been successfully used in a variety of different organisms [e.g., (Buma and Roubos, 1986; Morris and Pow, 1991)]. We focused on the arborizations around the DM/VM tract and especially the VL fibres in the ventral ganglion, as these structures appeared to fit the least into the classic textbook scheme of a compartmentalized neuron.

### Immuno-electron microscopic synapse analysis

#### Arborizations around the DM/VM tract in the ventral ganglion

**Figure 10:**
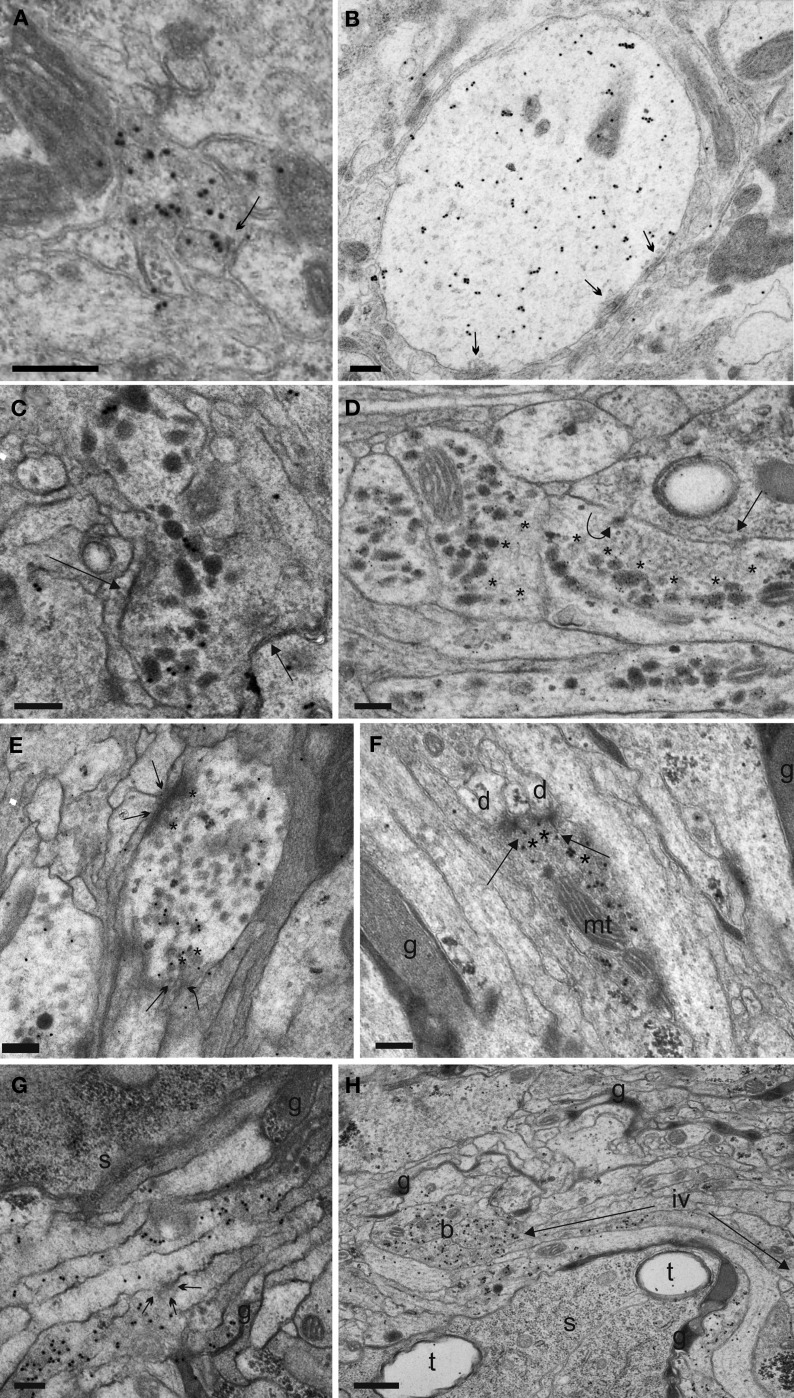
**Synaptic events and fibre morphology of the IN neurites in the DM/VM region (A,B) and ventrolateral (VL) tract (C–H) of *Drosophila* L3 larvae. (A)** One of the rare preparations of the DM/VM neuropile in which sufficient immuno-labeled DCVs could be preserved to identify the small N_CCAP_ fibers in this region. Arrow indicates a synaptic site between two labeled profiles. **(B)** Thick, dendrite-like N_CCAP_ fibre in the region of the DM/VM tract. Multiple output synapses with SSV are visible (arrows). The CAPA-immunoreactive DCVs are smaller as in the VL tract, and located far away from the synaptic surfaces. **(C)** An IN ventrolateral fibre portion gives two single synapses (arrows) with SSV exclusively. Immunogold grains label ectopically expressed CAPA peptides in DCVs. **(D)** CAPA-immunoreactive (immunoreactive) profiles of abdominal INs. Note large and separated accumulations of clear vesicles (asterisks). The arrow labels a single output synapse with small clear vesicles. The curved arrow marks a dense vesicle attached to the plasma membrane indicating non-synaptic peptide release. **(E)** Varicosity identified by CAPA-immunoreaction with two output synapses (arrows). The accumuled SSV on the presynaptic site are surrounded by immunolabeled DCVs (asterisks), which suggests possible paracrine DCV release. **(F)** CAPA-labeled profile with T-bar output synapses (arrows) onto small dendritic profiles (d). Note mass of small clear vesicles crowded in the cytoplasm (asterisks). g, glial processes; mt, mitochondrion. **(G)** A bunch of IN fibres running in the VL tract. Arrows show a microvesiculation event, an indicator of neurosecretory activity (Pow and Morris, 1991; Wasmeier et al., 2005). s, cell body; g, glial processes. **(H)** The VL tract in *Drosophila* larvae contains up to 13 CAPA-immunoreactive neurites. A single fibre divided in several large bead-like varicosities (b) growing thin joining intervaricose sections (iv, between arrows). t, tracheae; g, glial processes; s, soma. Scale bars = 200 nm **(A–F,H)**, 500 nm **(G)**.

The DM/VM region comprized thick neurites poor in dense granules, typically with several small synaptic profiles including SSV/presynaptic and postsynaptic sites in the thoracic and abdominal neuromeres (Figures 10A,B). Interestingly, around 60% of the observed synaptic events were formed between immunolabeled profiles, which suggests synaptic connections between the homotopic EN and IN. Due to the limited number of dense granules, the DM/VM profiles usually lacked immunlabeling and hence could be identified in a few samples only. It is therefore difficult to draw any general conclusion other than that the arborizations around the DM/VM tract in the thoracic and abdominal neuromeres contain presynaptic elements but do not represent a significant site of peptide release. This conclusion is also supported by the immunostaining pattern against the ectopically expressed CAPA peptide (not shown) and CCAP (Figure 8).

#### Immuno-electron microscopic synapse analysis of the VL fibres

**Figure 11:**
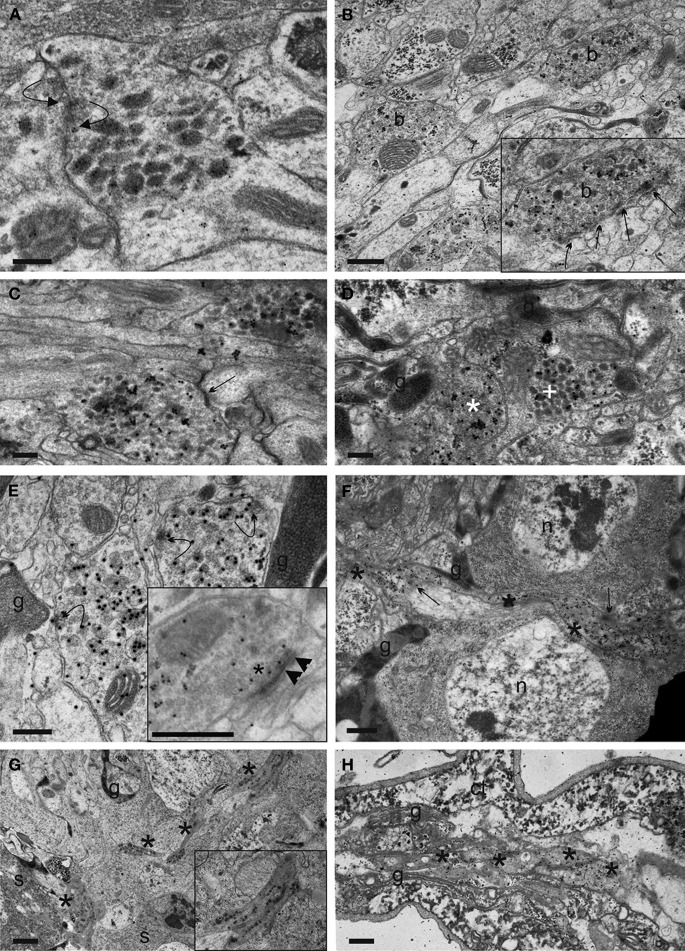
**Synaptic events and fibre morphology in IN neurites in the ventrolateral (VL) tract and transverse projections, and EN neurites in the segmental nerve of *Drosophila* L3 larvae. (A)** Reciprocal synapses with SSV agglomeration (curved arrows) between two IN neurites in the ventrolateral (VL) tract, identified by CAPA-immunoreactivity. **(B)** Portion of a VL tract fibre with two bead-like varicosities (b). Insert: Arrows mark serial output synapses made by the upper right varicosity in higher magnification. It is visible that the DCVs are distantly located from the synapses. **(C)** A SSV input synapse (arrow) of unknown identity onto a varicose section of a CAPA-labeled VL tract neurite. **(D)** IN profiles of different morphology in the thoracic part of the VL tract, identified by ectopic CAPA-peptide expression. Based on the frequency of morphological types, neurites can be putatively assigned to IN with somata in the thoracic or abdominal (white asterisks) or sog portion (white cross) of the ventral ganglion. g: glial processes. **(E)** Curved arrows show secretory granules attaching to or fusing to the plasma membrane at non-synaptic sites in CAPA-immunoreactive VL tract profiles. g: glial processes. The inset shows a different synaptic profile that indicates the rare event of synaptical DCV (arrow heads) intermingled with SSV (asterisk) at the active zone, possibly indicating synaptic peptide release. **(F)** Transverse fibre section (asterisks) running toward the ventral ganglion midline. Compared to VL tract neurites, this fibre type shows less prominent thickenings and more uniform DCV dispersion and immunolabeling. Arrows mark putative transmitter or peptide liberation areas, g, glial processes; n, nucleus. **(G)** Part of the dorsolateral region of the ventral ganglion. Asterisks mark sections of a CAPA-labeled putative abdominal EN neurite. Part of a thick neurite is enlarged in the insert. g, glial process; s, somata. **(H)** Asterisks mark portions of an EN neurite in a longitudinal section of an abdominal segmental nerve. Note the dense appearance of the cytoplasm in both **(G)** and **(H)** in the CAPA-immunoreactive structures. ct, connective tissue sheath; g, glial processes. Scale bars = 200 nm **(A–E)**, 500 nm **(F,H)**, 1 μm **(G)**.

**Figure 12:**
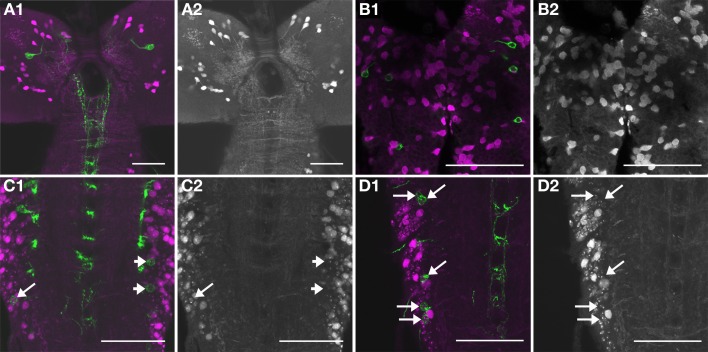
**Immunofluorescent stainings against GABA**. GABA immunoreactivity shown alone in gray **(A2,B2,C2,D2)**, or (in magenta) overlay with Ccap-Gal4-driven expression of GFP (green, **A1,B1,C1,D1**). In the brain [**(A1,A2)**, projection of 8 confocal slices containing the IN_brain_] and the sog [**(B1,B2)**, projection of 2 confocal slices containing the IN_sog_], all N_CCAP_ are GABA-immunonegative. In some but not all preparations of the thoracic and abdominal neuromeres, GABA immunoreactivity was found in one N_CCAP_ per hemineuromere. **(C1,C2)** While the N_CCAP_ on the right side of a4 and a5 are GABA-immunonegative (arrowheads), there is one N_CCAP_ on the left side in a5 that shows weak but distinct GABA labeling (arrow, projection of 2 confocal slices). **(D1,D2)** shows a projection of 8 confocal slices of another preparation, where the N_CCAP_ in a1, a3 and the anterior N_CCAP_ in a4 appear to be GABA-immunonegative (upper arrows). One of the N_CCAP_ in a4 (lowermost arrow) however shows weak GABA immunoreactivity. Scale bars = 50 μm.

After improving the aldehyde-tannic acid fixation protocol, we were able to identify synapses and follow relatively long consecutive sections of VL fibres in serial immunolabeled ultrathin sections (Figure S1). The immunoreactive processes showed large, bead-like varicose regions interspersed by small intervaricose sections. In total, we analysed 537 immunoreactive varicosities together with neighboring intervaricose sections. Altogether, we could identify and evaluate 559 synaptic events involving clear SSV (Table 1; Figures 10C–H, 11). The occurrence of distinct SSV clusters was apparently restricted to VL neurites originating from thoracic and abdominal IN fibres. This indicates that the thoracic and abdominal IN employ a classic transmitter in parallel to their different peptides located in DCVs. Since at least a subset of N_CCAP_ expresses functional GABA receptors (Vömel and Wegener, 2007) and synaptic events were found between N_CCAP_, we tested whether the SSV cluster contain GABA by immunostaining. The N_CCAP_ in the brain and sog were never labeled in any of the preparations at varying concentration of primary and secondary antiserum (Figures 12A,B), while many other neurons were strongly GABA-immunoreactive in a distinct pattern (Figure 12). Also most thoracic and abdominal N_CCAP_ where GABA-immunonegative, suggesting that the SSV in the IN do not contain GABA but rather an excitatory transmitter. Only a few thoracic and abdominal N_CCAP_ occassionally showed weak yet distinct GABA immunoreactivity. This GABA immunoreactivity in N_CCAP_ was spatially more restricted within the soma than in the more strongly labeled neurons, and confined to one of the two N_CCAP_ per abdominal hemineuromere. The staining in N_CCAP_ is unlikely to represent unspecific nuclear labeling, as most other N_CCAP_ in the same preparation were GABA immunonegative (Figures 12C,D). Instead, the weak and non-consistent GABA labeling could result from GABA uptake (Borden, 1996; Neckameyer and Cooper, 1998). It is however unclear whether N_CCAP_ express GABA transporters.

**Table 1:** **Types and distribution (in %) of synapses (*n* = 559) on the VL fibre**.

**Synapse type (*n* = 559)**	**Presynaptic**		**Postsynaptic**	
	**Bead**	**Intervaricose fibre**	**Bead**	**Intervaricose fibre**
Single synapse with SSV only	23.81	14.29	25.32	12.66
Single synapse with parasynaptic DCV release	19.05	10.47	3.79	10.13
Reciprocal synaptic profile	24.76	4.76	34.18	5.06
Serial synaptic element (only SSV)	2.86	0	8.86	0
Fraction (%) of total	70.48	29.52	72.15	27.85

Sites of non-synaptic peptide release are not included.

Another type of CAPA-immunoreactive fibres devoid of SSV and of different morphology intermingled with the SSV-containing neurites. These fibres most likely belong to the IN_sog2−3_. We often saw these distinct neurites closely apposed to the SSV-containing neurites, yet synaptic contacts were only rarely found between these two neurite types. Non-immunoreactive DCV-containing profiles also occurred in the proximity of immunoreactive fibres which may represent a further source of input onto N_CCAP_.

An ultrastructural synaptic analysis revealed that the neurites of the IN neurons in the ventral ganglion that run along the FasII-positive VL fibre (VL neurites) represent a mixed synaptic input-output compartment, as both pre- and postsynaptic sites were found (Table 1, Figures 10C–H, 11). This is in full agreement with the observed strong labeling of these neurites with both vesicle and dendritic fluorescent markers as detailed above. Summarized over both varicose and intervaricose sections, we obtained a ratio of 1.9:0.6:1 for output: reciprocal: input synapses (Figure 9A).

The presynaptic elements [SSV clustered at a membrane thickening (active zone)] of the VL fibre showed rather large presynaptic surfaces that often could be followed in a number of consecutive sections. For more then half of the individual output synapses (56.3%), a mass of DCVs was located extrasynaptically at sites without visible membrane specializations and in considerable distance from the active zone. DCVs formed larger aggregations within the varicosities, while only clear vesicles gathered at the presynaptic region (Figures 10C,D). For the other 43.7% of presynapses, DCVs occurred around the active zone within varicosities (Figure 10E). Thus, the spatial relation between DCV and SSV was typical for peptidergic neurons (Maley, 1990). A limited number of DCV were found to either be in close apposition to active zones in a juxta-synaptical position (in intimate contact with or attached to plasma membrane indicating peptide release), or para-synaptically released around the active zone (Figure 11E). Very rarely, a single labeled DCV was found within SSV aggregates at the active zone (Figure 11E). The common form of DCV release thus appeared to be non-synaptic at sites without membrane thickening and without SSV around (Figures 10D,G), while also parasynaptical release may occur. The non-synaptic DCV release could sometimes also be observed in transverse neurites running toward the midline (Figure 11F).

In intervaricose parts of the fibre, single DCVs or small clusters of max. 2–3 granules could be seen along filamentous elements. Yet, in contrast to varicosities (Figure 10H), DCV attachment to plasma membrane was a rare event. Input synapses were present in a few consecutive sections with usually little postsynaptic membrane differentiations. Typically, the postsynaptic part of these input synapses was seen as punctate specializations far from sites of DCV accumulations. Only a few prototype insect synapses with characteristic T-bars could be found (Figure 10F), all exclusively with SSV.

The majority of the observed synaptic elements were interconnected in an unidirectional fashion, but we also found a prominent cluster of reciprocal synapses both on varicosities and intervaricosities (18.35% of total, Table 1, Figure 11A). These reciprocal synapses were found between either immunoreactive and non-immunoreactive elements (67.7%), or between immunoreactive elements (32.3%). Most of immunoreactive structures were varicose elements or transition regions, and no DCV was found around the active zone.

The results suggest that the small-diameter intervaricose sections are not sites of peptide release. Surprisingly yet, they appear to play a considerable role in synaptic communication between VL fibre bundles and surrounding elements, while peptide release is largely restricted to varicosities. Intervaricose sections are involved as pre- or postsynaptic surfaces in 15.3% and 12.4% of the total synaptic elements, respectively.

### Imaging

**Figure 13:**
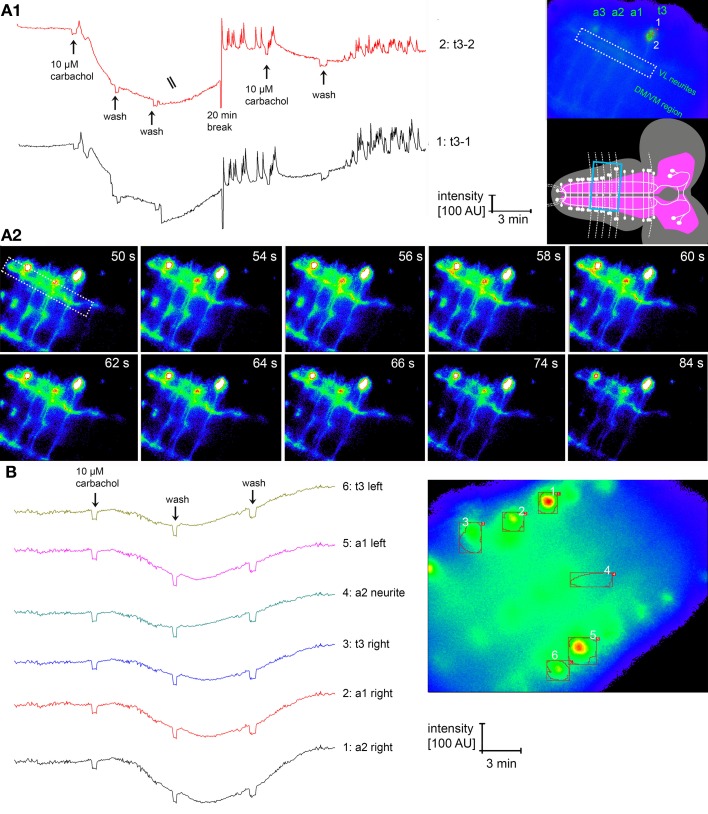
**[Ca2^+^]_i_ responses of N_CCAP_ in the intact dissected CNS upon application of the non-degradable AChR agonist carbachol. (A1)** Specific response of N_CCAP_ in t3 [region of interests (ROI) 1- and 2 labeled in the inset to the top right]. Directly after carbachol application, a small brief [Ca^2+^]_i_ increase is visible in both t3 cells, followed by a strong and long-lasting [Ca^2+^]_i_ decrease. After a wash and 20 min break, [Ca^2+^]_i_ oscillations occurred. The same pattern could be induced by a second application of carbachol. It is clear that both neurons show [Ca^2+^]_i_ responses that appear to be very similar. Yet widefield microscopic imaging does not allow to fully separate the fluorescence information of the two cells, thus the fluorescence signals overlap to some degree. It is also in general not possible to tell apart IN and EN when GCaMP1.6 is expressed in all N_CCAP_. The inset bottom right shows the visual field during the experiment, boxed in blue. Besides the t3 cells, also the a1–3 cells of the same side and corresponding VL and transversal neurites are visible. **(A2)** Single imaging frames from the experiment in **(A1)**, 50–84 s after application of carbachol, showing a calcium wave running through the VL fascicle (boxed, for overview see **A1** right) at 56–64 s, followed by the onset of a [Ca^2+^]_i_ decrease. The false color of the frames is overenhanced in comparison to **(A1)** to better visualize the small changes in [Ca^2+^]_i_ in the neurites. The [Ca^2+^]_i_ decreases in the somata are partly masked by the overenhancement. **(B)** The typical response of the N_CCAP_ to carbachol is a strong [Ca^2+^]_i_ decrease that is not coupled to subsequent [Ca^2+^]_i_ oscillations or increase. While oscillations were observed in many N_CCAP_ in t3 (see **A1**), some only reacted with a [Ca^2+^]_i_ decrease (see **B**).

**Figure 14:**
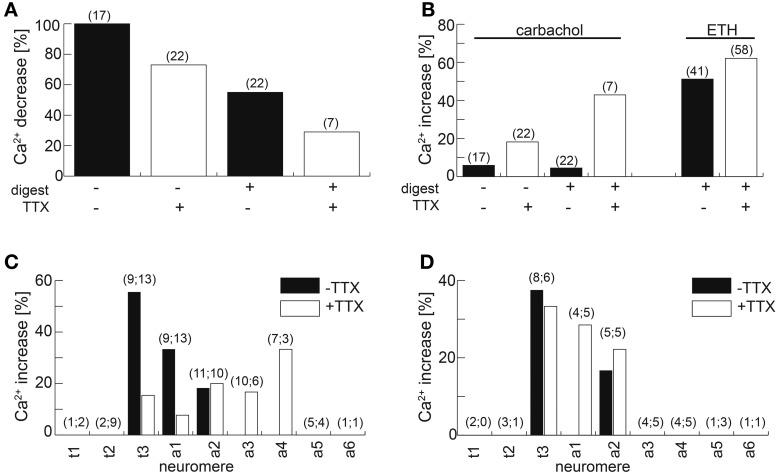
**Calcium responses of N_CCAP_ to the AChR agonist carbachol and ETH. (A)** Fraction of cells responding with a [Ca^2+^]_i_ decrease upon application of 10 μM carbachol. Fraction size is decreased by the presence of 100 nM TTX in preparations with or without prior enzyme treatment. **(B)** Fraction of cells responding with a [Ca^2+^]_i_ increase/oscillations upon application of 10 μM carbachol or 100 nM ETH-1.Fraction size is increased by the presence of 100 nM TTX in preparations with or without prior enzyme treatment. **(C)** Fraction of N_CCAP_ responding to 100 nM ETH in wandering 3rd instar larvae. The neuromere location of the neurons is indicated on the x-axes. Adding 100 nM TTX broadened the neuromere distribution of responding N_CCAP_. **(D)** Fraction of N_CCAP_ responding to 100 nM ETH in feeding 3rd instar larvae. The neuromere location of the neurons is indicated on the x-axes. Adding 100 nM TTX did not broaden the neuromere distribution of responding N_CCAP_. Number in brackets indicates *n*.

Our anatomical results on the single cell and ultrastructural level open the possibility of a considerable reciprocal synaptic cross-talk between the IN in the ventral ganglion. Previous reports had shown that larval N_CCAP_ express functional acetylcholine receptors (AChRs), with N_CCAP_ subsets expressing functional receptors for ETH and the transmitters glutamate and GABA (Kim et al., 2006; Vömel and Wegener, 2007). We therefore set out to functionally test whether the reciprocal synapses may underlie a mechanism of IN synchronization throughout the ventral ganglion. By *in situ* imaging we recorded the [Ca^2+^]_i_ responses of the strongly GCaMP-expressing N_CCAP_ in t1–3 and a1–6 upon bath-application of 10 μM carbachol (a non-degradable AChR agonist) in intact CNS of wandering 3rd instar Ccap-Gal4xUAS-GcAMP1.6 larva. Typical [Ca^2+^]_i_ responses to carbachol are shown in Figure 13. In contrast to our expectation but in line with previous results (Vömel and Wegener, 2007), carbachol induced a slow decrease in [Ca^2+^]_i_ in all tested neurons (Figure 14A), that in many preparations were followed by calcium oscillations in putative EN in t3 and a1. This lack of a general carbachol-induced activity did not allow to test whether the IN show synchronized [Ca^2+^]_i_ responses.

ACh, nicotine and the muscarinic AChR agonist pilocarpine induced [Ca^2+^]_i_ increases in cultured N_CCAP_ (Vömel and Wegener, 2007) rather than decreases. We next tested whether the observed carbachol-induced [Ca^2+^]_i_ decrease *in situ* is due to indirect synaptic inhibition. Blocking synaptic activity by bath-applied 100 nM TTX reduced the fraction of N_CCAP_ responding with a [Ca^2+^]_i_ decrease to 73%. When the preparations were gently treated with enzymes to enhance tissue permeability prior to bath-applicating the drugs, a [Ca^2+^]_i_ decrease was observed in only 29% of the cells tested. Enzyme treatment alone reduced this fraction to 58% (Figure 14A). Without TTX, oscillating [Ca^2+^]_i_ increases were only observed in 5–6% of the tested N_CCAP_ (Figure 14A), all situated in t3 and a1 independent of enzyme treatment. This fraction could be tripled by adding TTX, and further increased to 43% when TTX and enzyme treatment was combined (Figure 14B). This suggests that synaptic inhibition is at least partially underlying the carbachol-induced [Ca^2+^]_i_ decrease. The TTX diffusion into the CNS may also be limiting its potential blocking effect.

**Figure 15:**
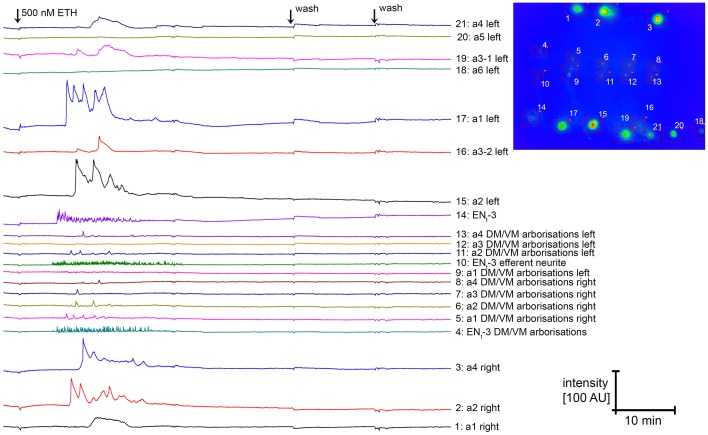
**[Ca^2+^]_i_ responses of N_CCAP_ to application of the peptide ETH-1**. All neurons with a [Ca2^+^]_i_ response are located in neuromeres t3 or a1-a4. It is visible that the [Ca2^+^]_i_ response of neurons of a given neuromere pair are not synchronized. Since it is in general not possible to tell apart IN and EN when GCaMP1.6 is expressed in all N_CCAP_, it is unclear whether the responding cells represent IN or EN. In this and few other preparations, EN_t3_ could however be identified due to the efferent neurite, and shows [Ca^2+^]_i_ oscillations which are typical for this cell type. The inset to the right indicates cell position and ROIs.

Since carbachol application did not lead to an activation of N_CCAP_, we next applied 500 nM ecdysis-triggering hormone (ETH) to induce synchronized activation. Typical [Ca^2+^]_i_ responses to ETH are shown in Figure 15. In contrast to carbachol, ETH induced a [Ca^2+^]_i_ increase in half of the tested unassigned N_CCAP_ in enzyme-treated preparations, a fraction that only slightly increased to 62% in the presence of TTX (Figure 14B) which is in line with a direct effect. TTX application also did not discernibly affect the time between ETH application and [Ca^2+^]_i_ response, which was highly variable and typically ranged from 10–30 min (Figure 15). We repeated this experiment with CNS from wandering 3rd instar larvae, now with identified neuromeres (Figure 14C). This revealed that only N_CCAP_ in t3 and a1–2 responded to ETH in the absence of TTX. With TTX, also neurons in a3–4 became activated, though fewer N_CCAP_ in t3 and a1 responded (Figure 14C). In feeding larvae, which have not yet seen the peak of the steroid hormone ecdysone that induces wandering behavior (Warren et al., 2006), N_CCAP_ in t3 and a2 (without TTX) or in t3 and a1–2 (with TTX) showed a [Ca^2+^]_i_ response upon ETH application (Figure 14D). This suggests that ETHR expression in the N_CCAP_ is not as tightly linked to ecdysteroid titres as in other insects (Zitnan et al., 2007; Dai and Adams, 2009). Nevertheless, we could not detect synchronization of the [Ca^2+^]_i_ responses among the responding neurons (most likely ENs), including contralateral pairs of the same neuromere. Though not fully conclusive, the results point to a prevailing inhibitory synaptic input to the N_CCAP_ which appears to be extrinsic as N_CCAP_ are lacking a clear GABA immunosignal. This inhibitory input likely can be activated by carbachol, and may account for at least a part of the input synapses onto the N_CCAP_.

## Discussion

### The N_CCAP_ can be grouped into four classes with a distinct polarity and complexity

Our single cell labelings show that the larval N_CCAP_ can be grouped into five different neuron classes. (1) local interneurons in the protocerebrum (IN_brain_-1), (2) projection neurons with somata in the protocerebrum (IN_brain_-2), (3) projection neurons in the ventral ganglion (IN_sog2−3_, IN_t_, IN_a_), (4) local interneurons in the ventral ganglion (IN_sog1_), and (5) efferent neurons in the ventral ganglion (EN_sog2_, EN_t3_, EN_a1−4_).

Based on the distribution of expression markers and peptide immunoreactivity plus varicose vs. smooth neurite morphology, IN_brain_ neurons (group 1–2) can be regarded as polarized neurons in terms of in- and output sites. They apparently have separated dendritic input- and axonal output compartments. The EN (group 5) have an axonal output compartment at the body wall muscles (Vömel and Wegener, 2007). Due to the overlap of their central arborizations with group 4 neurites around the midline of the ventral ganglion, it is, however, difficult to assess whether the central arborizations of the EN represent a site with purely dendritic input- or mixed non-peptidergic in- and output. Group 4 neurons are not clearly polarized since both vesicle- and dendritic markers overlap considerably along the neurites with possible exception of the distal-most fine arborizations around the DM-VM tract that were only labeled by DenMark. Since DenMark gave in general a much more intense fluorescence signal than the GFP-tagged vesicle markers, we can, however, not exclude that we have overlooked the vesicle marker labeling of the fine arborizations.

The marker-based designation of peptidergic output compartments of the N_CCAP_ in the ventral ganglion is in full agreement with the loss of fluorescence of CCAP- or bursicon immunoreactivity or an ectopically expressed GFP-tagged peptide reporter (ANF-EMERALD) in N_CCAP_ from both type III neurohaemal terminals on the body wall and the VL fibre during larval ecdysis (Park et al., 2003; Husain and Ewer, 2004; Loveall and Deitcher, 2010; Lahr et al., 2012).

### The polarity of the ventral ganglion IN is hardly definable in terms of dendrites and axons

The co-occurrence of strong labeling for peptides, vesicle- and dendritic markers in the VL fibre is fully supported by the electron-microscopic data that showed that the group 4 (IN_sog2−3_, IN_t_ and IN_a_) neurites possess a remarkably complex local circuitry along the VL tract and contain pre, post- and reciprocal synaptic elements as well as non-synaptic peptide release sites. This match between fluorescent labeling and immuno-EM suggests that the distribution of the ectopically pre- and postsynaptic markers as well as CAPA prepropeptide is not significantly affected by the GAL4-directed over-expression of the genetic markers.

In vertebrates, peptide and neurotransmitter release from dendritic compartments is well documented (see Ludwig and Pittman, 2003; Ludwig and Leng, 2006), as are dendro-dendritic and axo-axonic synapses onto peptidergic neurons (e.g., Silverman et al., 1983; Silverman and Witkin, 1985; Guan et al., 2003). In insects and other invertebrates however, dendritic arborizations arise from stem branches, similar to axonal branchings. It is therefore hard to say whether the group 4 neurites along the VL tract represent a dendritic or axonal compartment, even though neurons with distinguishable axon and dendrites exist in *Drosophila* (see Rolls, 2011) and dendritic and axonal compartments can be assigned to most N_CCAP_ based on the distribution of vesicle and dendritic markers. The occurrence of presynaptic elements on dendritic structures or postsynaptic elements on axons are well documented for insect neurons (see introduction), though typically either pre- or postsynaptical sites predominate. For peptidergic neurons, the occurrence of pre- and postsynaptic elements on the same section have been reported for “dendritic” branches of PDF-expressing neurons in the accessory medulla of flies (Yasuyama et al., 2006) and cockroaches (Reischig and Stengl, 2003), and in the “axonal” branches in the dorsal protocerebrum of the fly (Yasuyama and Meinertzhagen, 2010). Along the VL neurites, the number of output synapses is only twice that of input synapses, compared to e.g., a ratio of 10:1 in the PDF neuron branches in the dorsal protocerebrum (Yasuyama and Meinertzhagen, 2010). In projection neurons (and obviously also the IN_brain_), dendritic branches are often located closer to the soma than the axon terminals, which would argue that the small arborizations in the ipsilateral neuropile represents the IN dendrites, while the arborizations around the midline as well as the VL neurite would represent axonic compartments giving only synaptic or synaptic and peptidergic output while receiving axo-axonic synaptic input. This would fit with the higher number of pre- than postsynaptic elements in the VL neurites. If correct, then UAS-DenMark, UAS-shal2 and UAS-Dscam17.1 represent postsynaptic rather than dendritic markers in the fruitfly.

Most postsynaptic specializations in VL neurites were concentrated on varicosities or in the transition regions between varicose and non-varicose sections. In accordance, we found that the postsynaptic membrane marker labeling was significantly higher on and in the close vicinity of varicosities at high magnifications in the confocal microscope. This suggests that the varicosities are the main location of pre- and also postsynaptic communication in addition to being the preferred site of peptide liberation. This finding is in contrast to results from serial section electron microscopy of a larval *Drosophila* CNS, which suggest that postsynaptic sites are almost exclusively localized to thin branches of varicose neurites (Cardona et al., 2010). Also in locusts, synapses are mostly found on small-diameter neuropilar branches (Watson and Schürmann, 2002). While we cannot fully exclude that we have overlooked such thin branches, our results nevertheless show that also varicosities can form postsynaptic events. The continuous postsynaptic marker protein labeling along the entire VL fibre at lower magnification in the confocal microscope can be explained by the number of IN neurites that can even branch into smaller elements within the VL fibre. Varicosities are juxtapositioned unevenly along these neurite bundles resulting in a relatively uniform signal strength and fibre bundle thickness.

Taken together, our observations indicate that VL neurites are not compartmentalized in clearly spatially separated in- and output sectors and thus do not comply with the typical pattern found in most non-peptidergic insect neurons (Cardona et al., 2010). Also the observed monodiadic or reciprocal synapses lacking T-bars deviate from the standard fly pattern of a polyadic T-bar synapse (Prokop and Meinertzhagen, 2006; Cardona et al., 2010). Rather, evenly located varicosities appear to act as “communication centres” with highly complex local synaptic circuitry which in principle could allow for localized peptide release from only a part of a neurite. Alternatively, the largely non-overlapping occurrence of varicosities on different neurites within a VL tract could serve to establish a uniform coverage of synaptic events and peptide release throughout the ventral ganglion. Nonetheless, the occurrence of synaptic events in intervaricose regions suggest that also these non-varicose sections may play a role as a place of synaptic communication.

It is important to stress that the IN in the thoracic and abdominal ganglia obviously do not only have mixed synaptic and peptidergic output at the VL neurites, but also seem to possess a predominantly synaptic output compartment in the arborizations around the DM/VM tract. This suggests that peptidergic neurons can use region-specific ways of signaling depending on the neuritic compartment. This need to be taken into account in connectomics studies and cautions against simple wiring diagrams of peptidergic neurons based on peptide distribution only.

### The N_CCAP_ release their peptides in a non-synaptic and parasynaptic mode from the VL neurites

Captured events of DCV release or intimate DCV contact to the plasma membrane was largely confined to varicosities and was mostly observed at non-synaptic sites, but occurred also in a considerable number at parasynaptic sites. This argues against a spatially restricted “co-transmitter” release of the N_CCAP_ peptides from the VL neurites, and is in favor of paracrine or volume transmission. Our data does not allow to assess the spatial extent of this non-synaptic transmission: this will require a receptor mapping in the future to identify the target structures.

### Reciprocal synapses between the IN in the ventral ganglion may coordinate fast and system-wide peptide release

Genetical ablation of the N_CCAP_ in the *Drosophila* larva does not impair the general execution of pre-ecdysis and ecdysis behavior, yet specifically prolongs the ecdysis phase from about 1 to 3 min (Park et al., 2003). However, when both N_CCAP_ and neurons expressing eclosion hormone (EH) were co-ablated, this resulted in a significantly increased impairment of pre- and ecdysis-behavior that was not observed when ablating either neuron type alone, including larvae that were unable to shed their old mouthparts (Clark et al., 2004). Thus, N_CCAP_ have an important function during larval ecdysis that appears to be in large part backed-up by EH.

Unlike the peripherally released peptides of the EN (Loveall and Deitcher, 2010), CCAP is released from the VL neurites of the IN in a very narrow time window starting around 3 min prior to larval ecdysis (Park et al., 2003). The observation of reciprocal synapses between IN neurites in the VL tract immediately suggests a testable mechanism that could synchronize the activity of these different neurites to coordinate and confine the surge of peptide release within a few minutes. Reciprocal synapses had, to our knowledge, not been described for any invertebrate peptidergic neuron. They occur, however, between peptidergic neurons in the synaptic feeding-regulating circuitry of the mammalian arcuate nucleus. Here, NPY- and ghrelin neurons form reciprocal axo-axonic synapses (Guan et al., 2003), while reciprocal axo-somatic synapses can be found between NPY and enkephalin-producing neurons (Li et al., 1993). Both NPY and enkephalin-producing neurons also form reciprocal autosynapses (Li et al., 1993). The resulting local feedback is thought to enable NPY neurons to self-modulate NPY release.

### Conflict of interest statement

The authors declare that the research was conducted in the absence of any commercial or financial relationships that could be construed as a potential conflict of interest.

## Supplementary material

Figure S1 **A stitched overview of a longer portion of the VL tract at the border of the abdominal and thoracic region**. Ellipse, bead-like varicose thickenings full of dense granules; dotted line, intervaricose junction; -p, CAPA-immunonegative DCV-containing fibres; arrows, putative synaptic connections; g, glial cell processes; sog, descending fibre of suboesophageal IN origin. Double arrows and stars mark a small intervening glial process isolating a non-immunoreactive and a CAPA-labeled IN process. Note non-overlapping occurrence of the single varicosities connected via thinner intervaricose sections. This kind of arrangement results in a by and large homogeneous VL tract diameter and a seemingly uniform intensity of immunofluorescent labeling in the confocal preparations. Presence of single, clearly distinct varicosities were only occasionally appreciable by confocal microscope in non-flpout animals. Scale bar = 200 nm.
