# Supplementary figures and images for: Diverse in- and output polarities and high complexity of local synaptic and non-synaptic signaling within a chemically defined class of peptidergic Drosophila neurons
Source: Front Neural Circuits. 2013 Aug 1;7:127. doi: 10.3389/fncir.2013.00127 (PMC3729985; doi:10.3389/fncir.2013.00127)

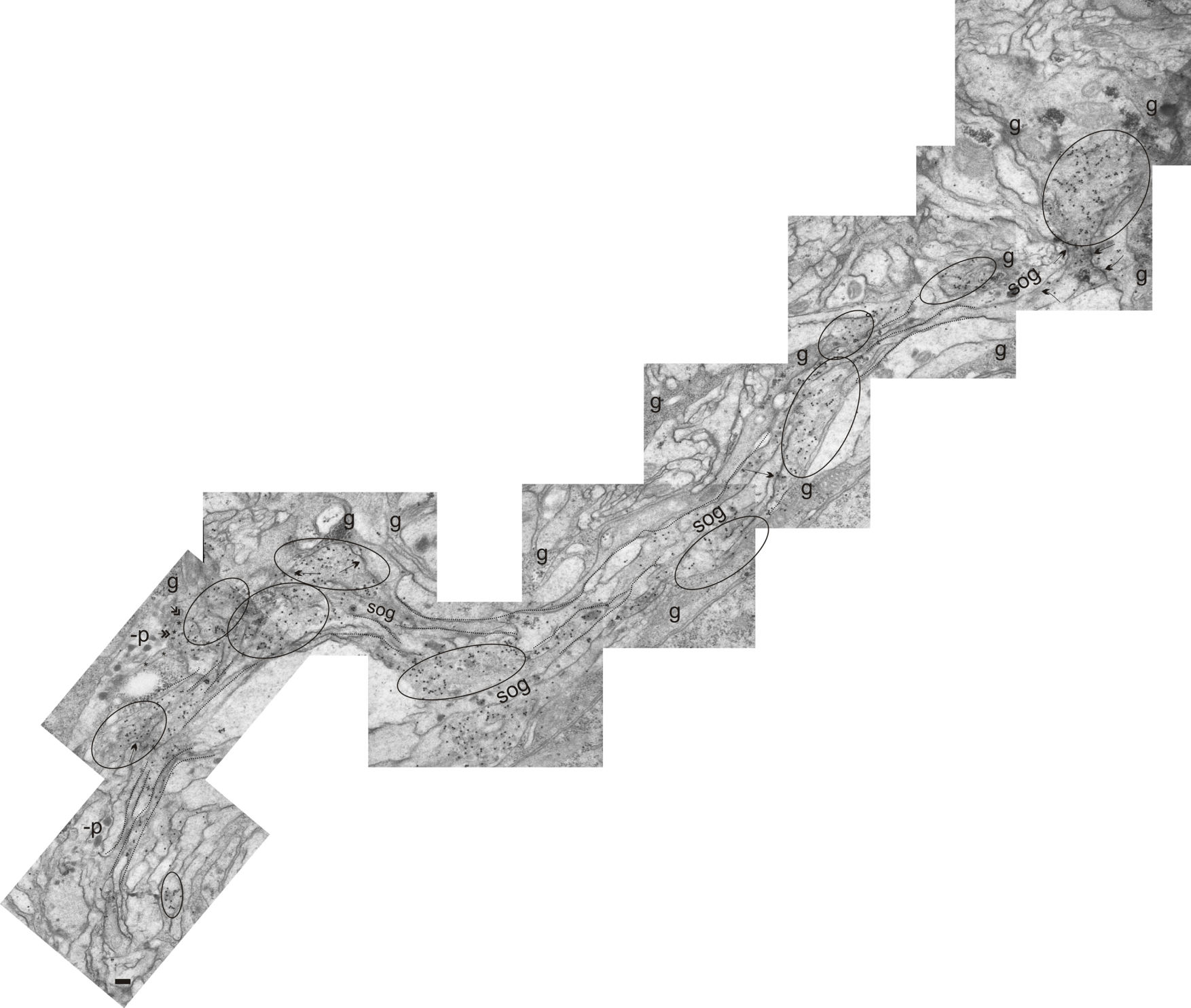

Supplement: Figure S1 — A stitched overview of a longer portion of the VL tract at the border of the abdominal and thoracic region. Ellipse, bead-like varicose thickenings full of dense granules; dotted line, intervaricose junction; -p, CAPA-immunonegative DCV-containing fibres; arrows, putative synaptic connections; g, glial cell processes; sog, descending fibre of suboesophageal IN origin. Double arrows and stars mark a small intervening glial process isolating a non-immunoreactive and a CAPA-labeled IN process. Note non-overlapping occurrence of the single varicosities connected via thinner intervaricose sections. This kind of arrangement results in a by and large homogeneous VL tract diameter and a seemingly uniform intensity of immunofluorescent labeling in the confocal preparations. Presence of single, clearly distinct varicosities were only occasionally appreciable by confocal microscope in non-flpout animals. Scale bar = 200 nm. [file Presentation1.PDF]
